# Supplementary material for: Comparative Proteomic Analysis of Wild and Cultivated Amaranth Species Seeds by 2-DE and ESI-MS/MS
Source: Plants (Basel). 2024 Sep 29;13(19):2728. doi: 10.3390/plants13192728 (PMC11478449; doi:10.3390/plants13192728)

Hydrophilic Fraction (H1) HR-2DE

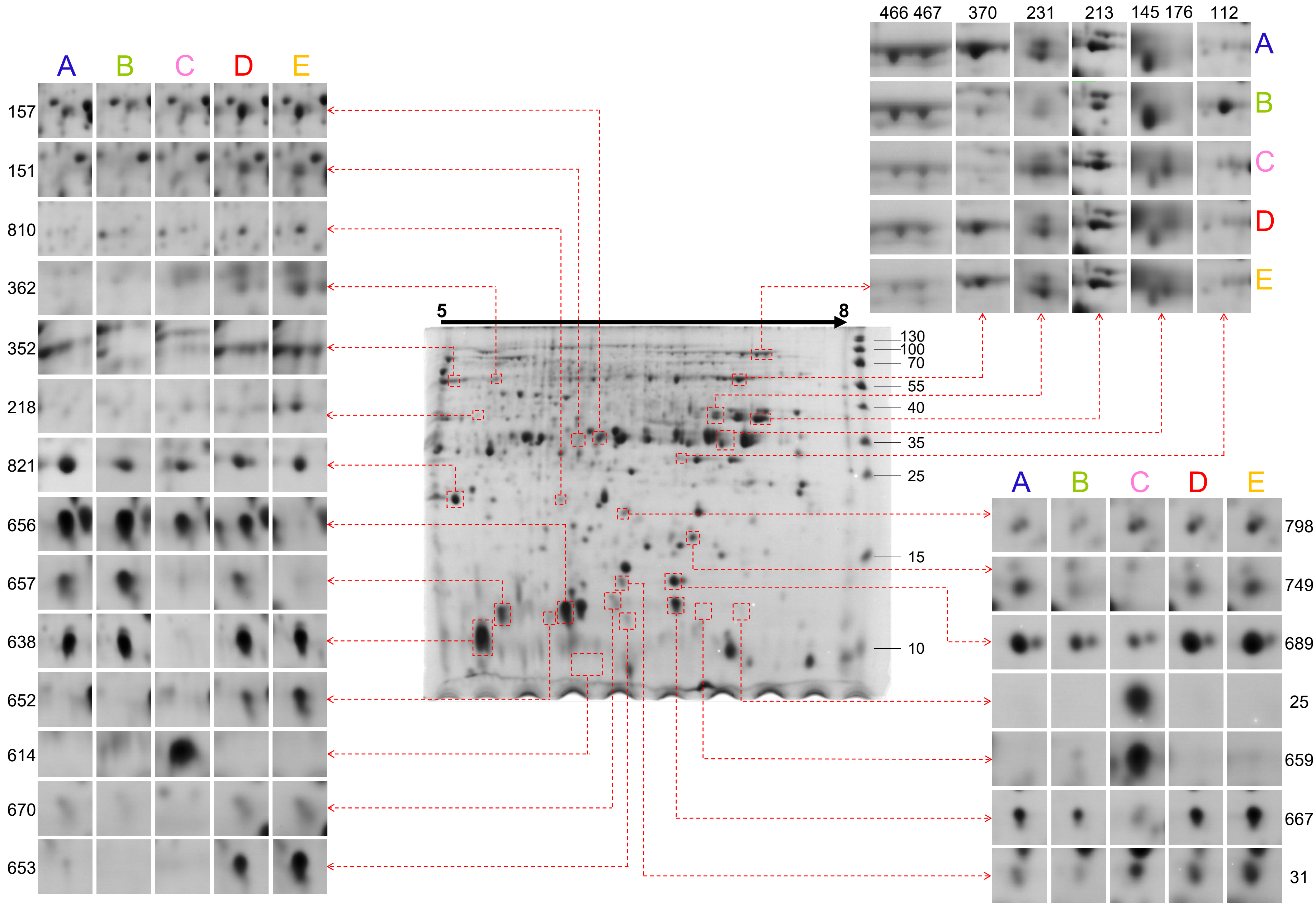

H1 HR-2DE Spot numbers

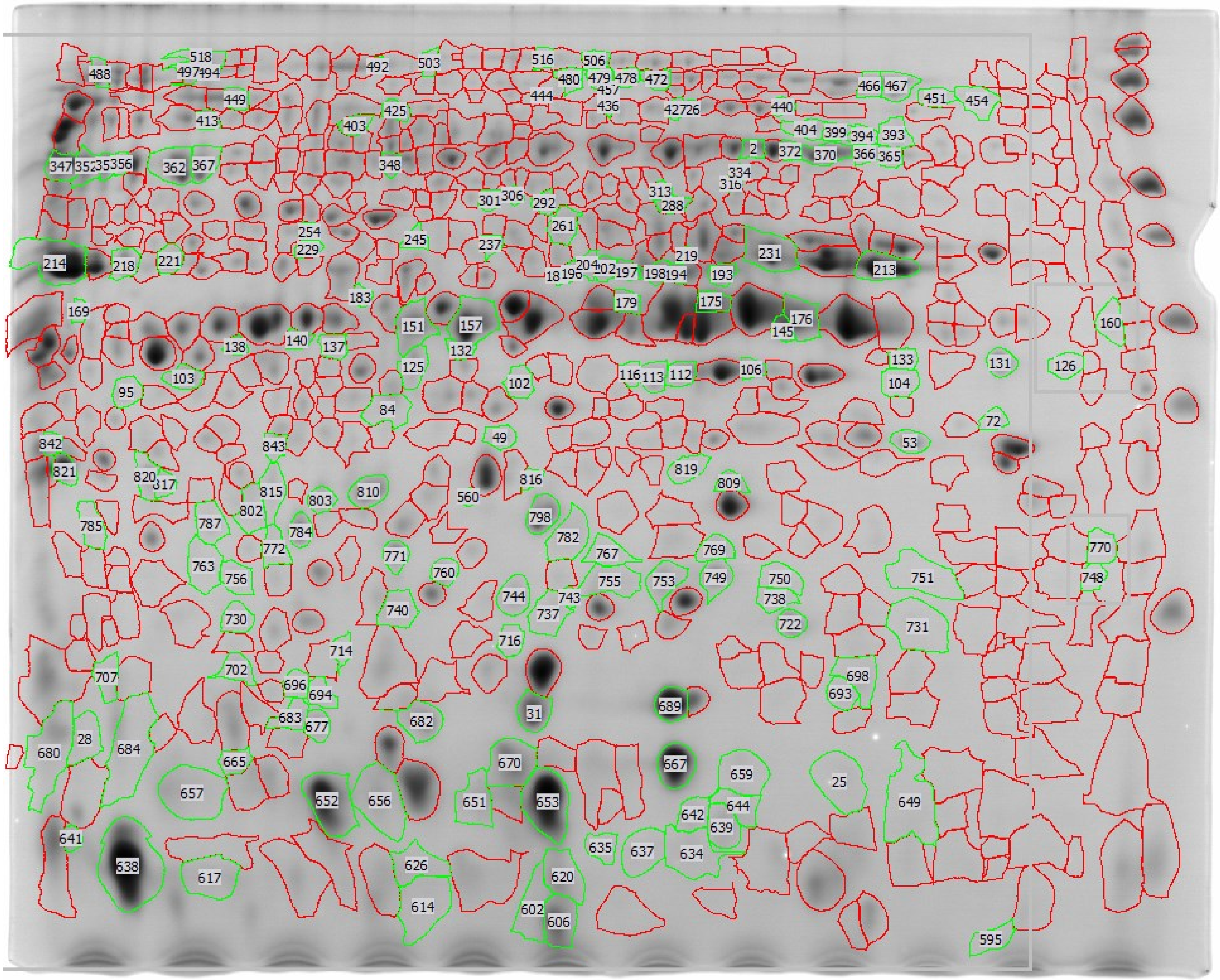

H1 HR-2DE Spot numbers Zoom 01

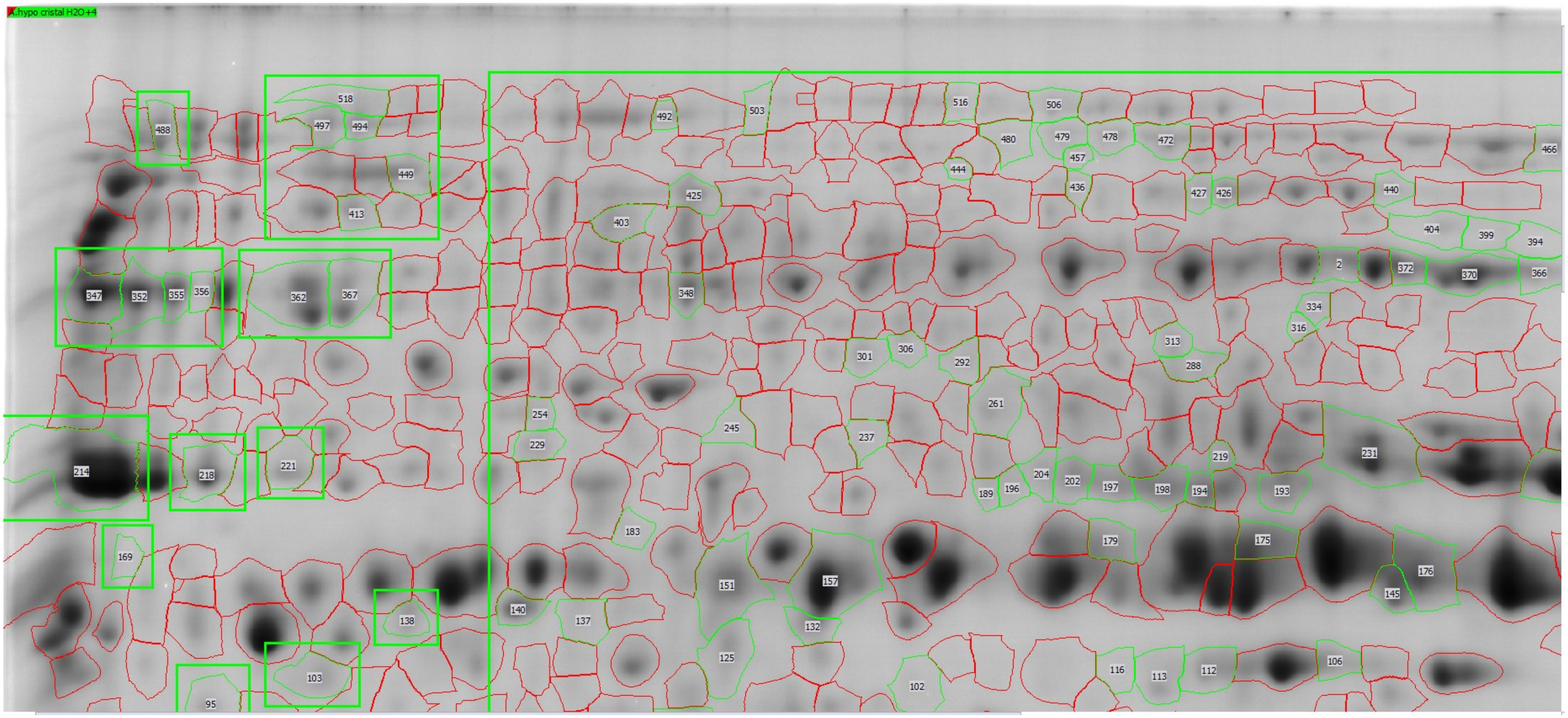

H1 HR-2DE Spot numbers Zoom 02

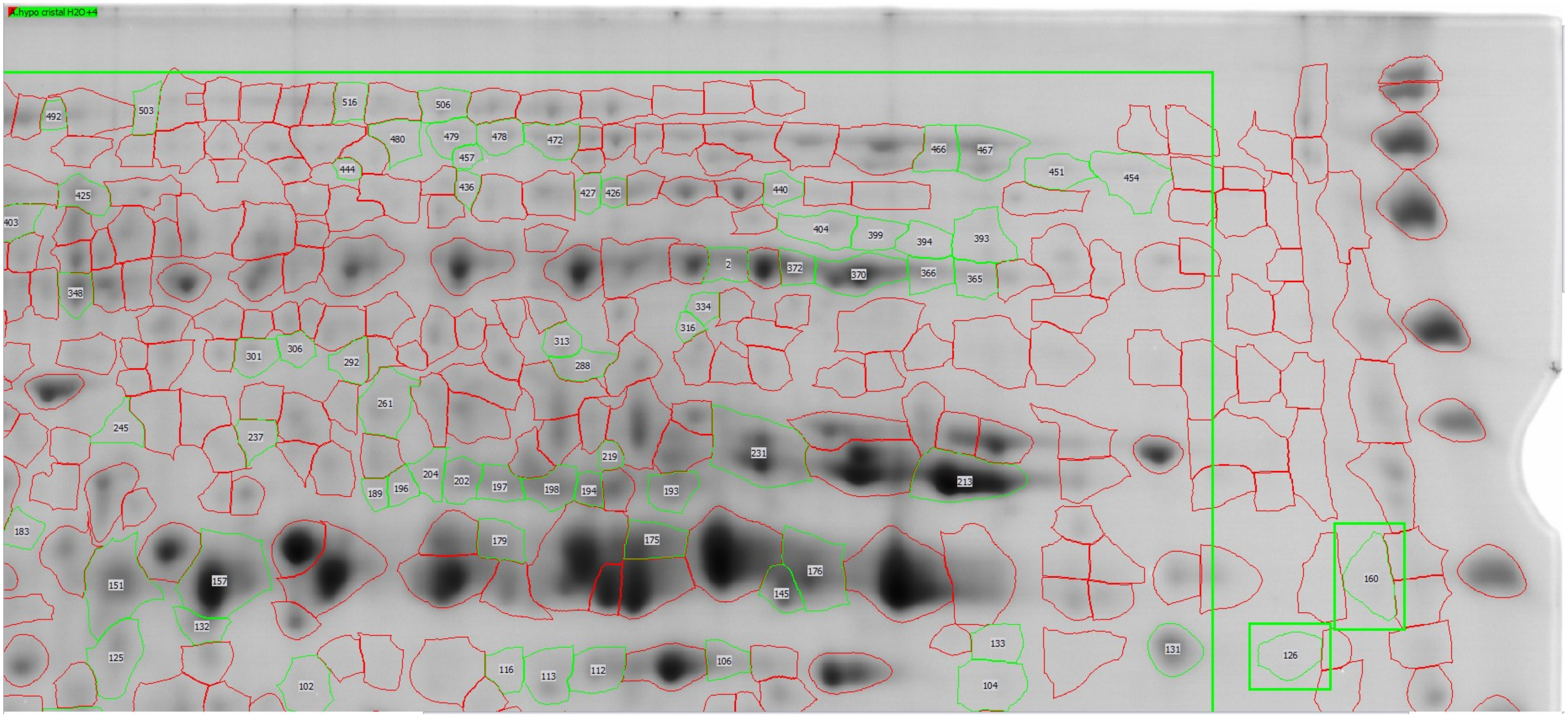

H1 HR-2DE Spot numbers Zoom 03

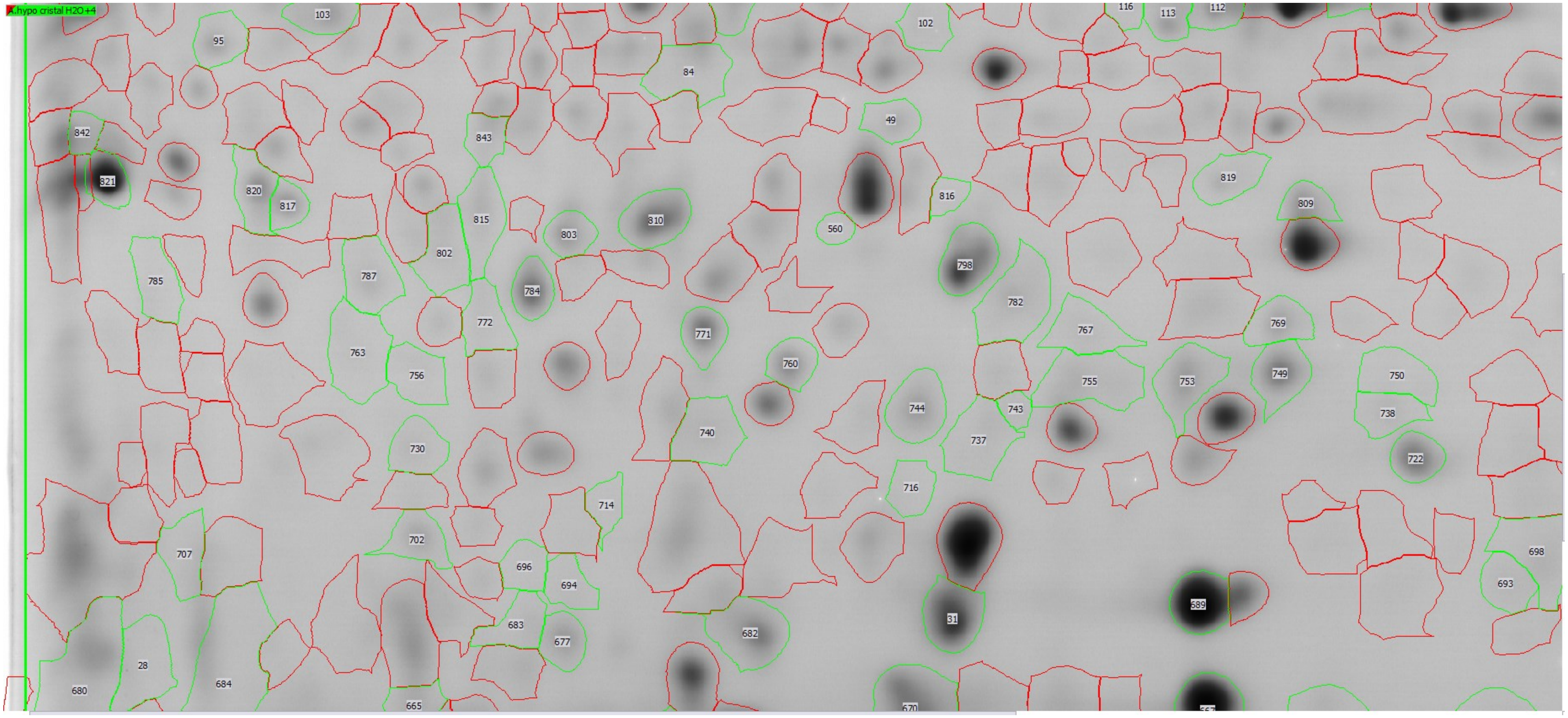

H1 HR-2DE Spot numbers Zoom 04

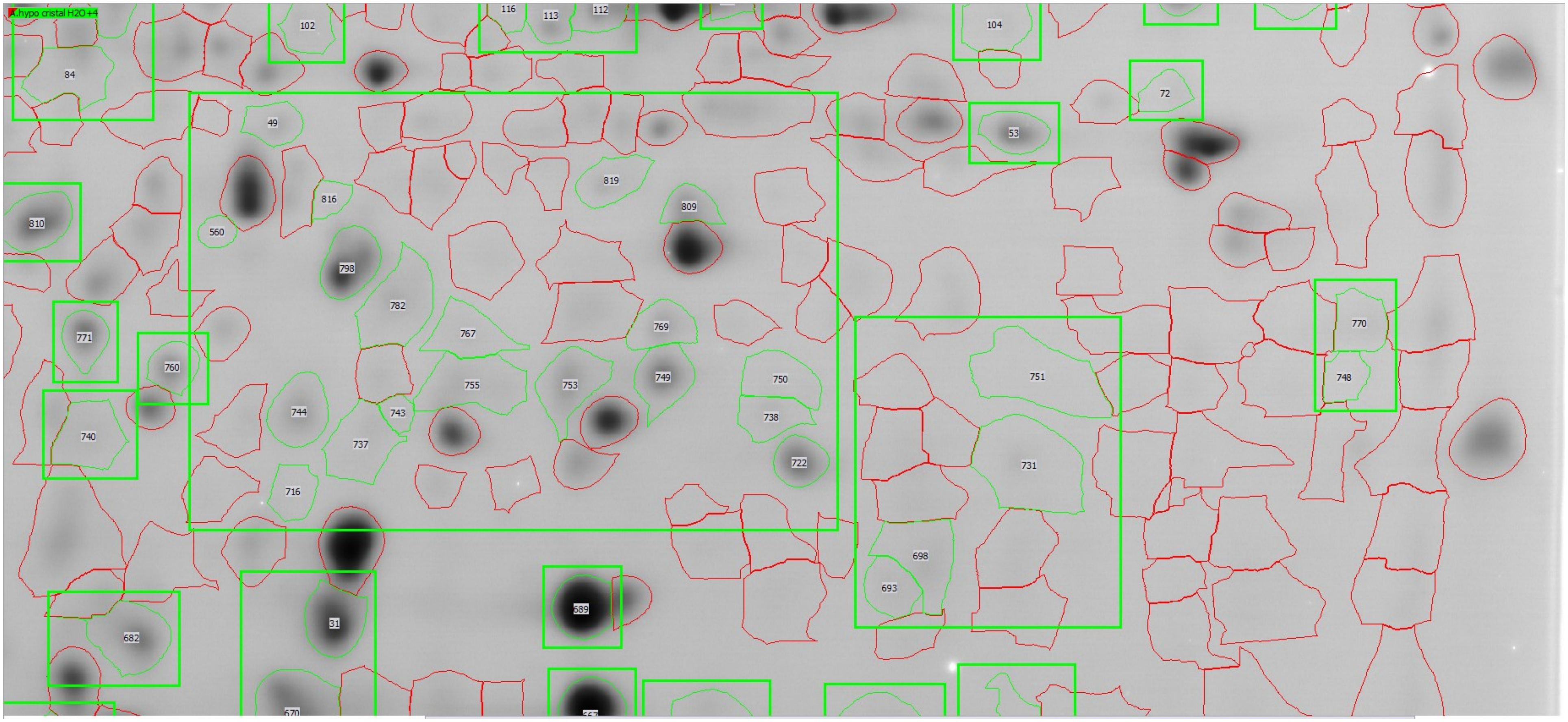

H1 HR-2DE Spot numbers Zoom 05

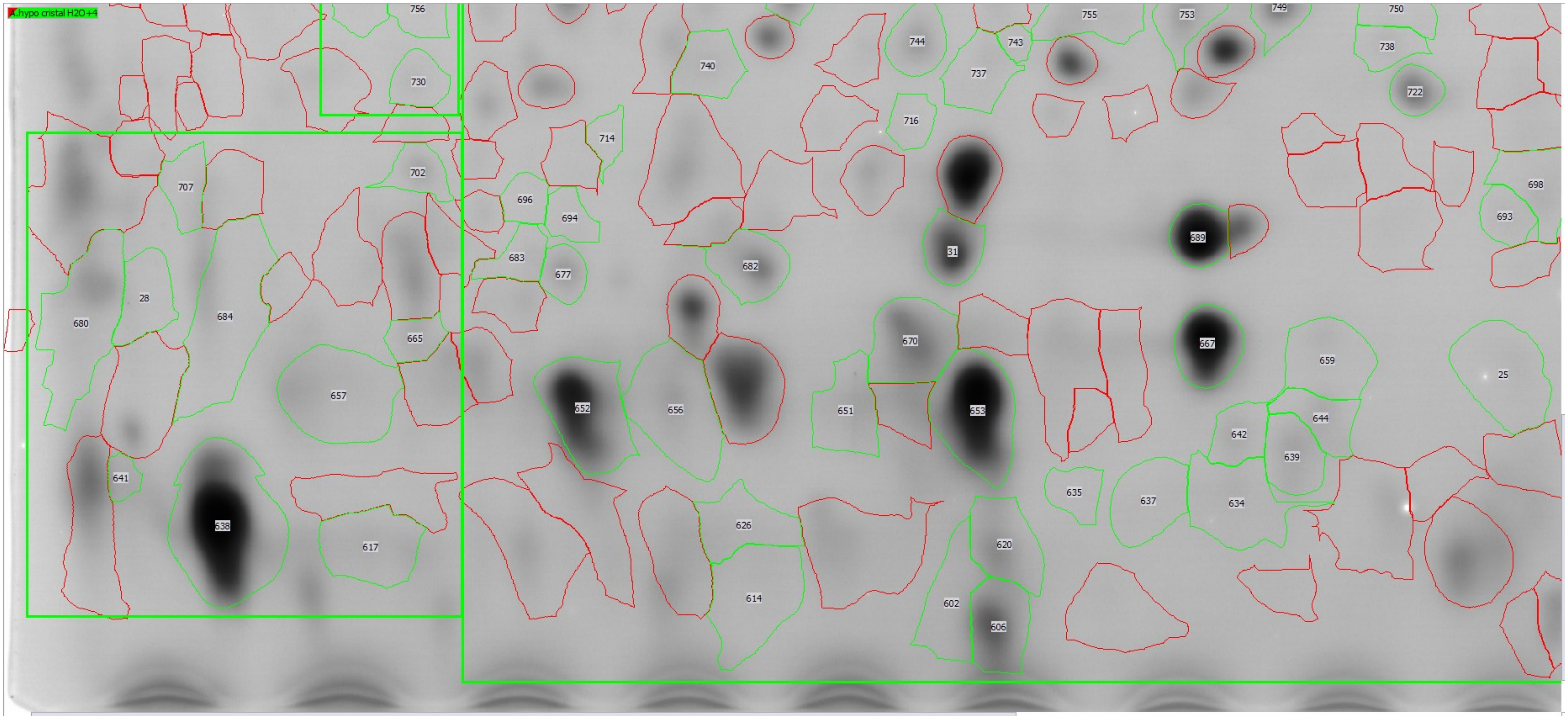

H1 HR-2DE Spot numbers Zoom 06

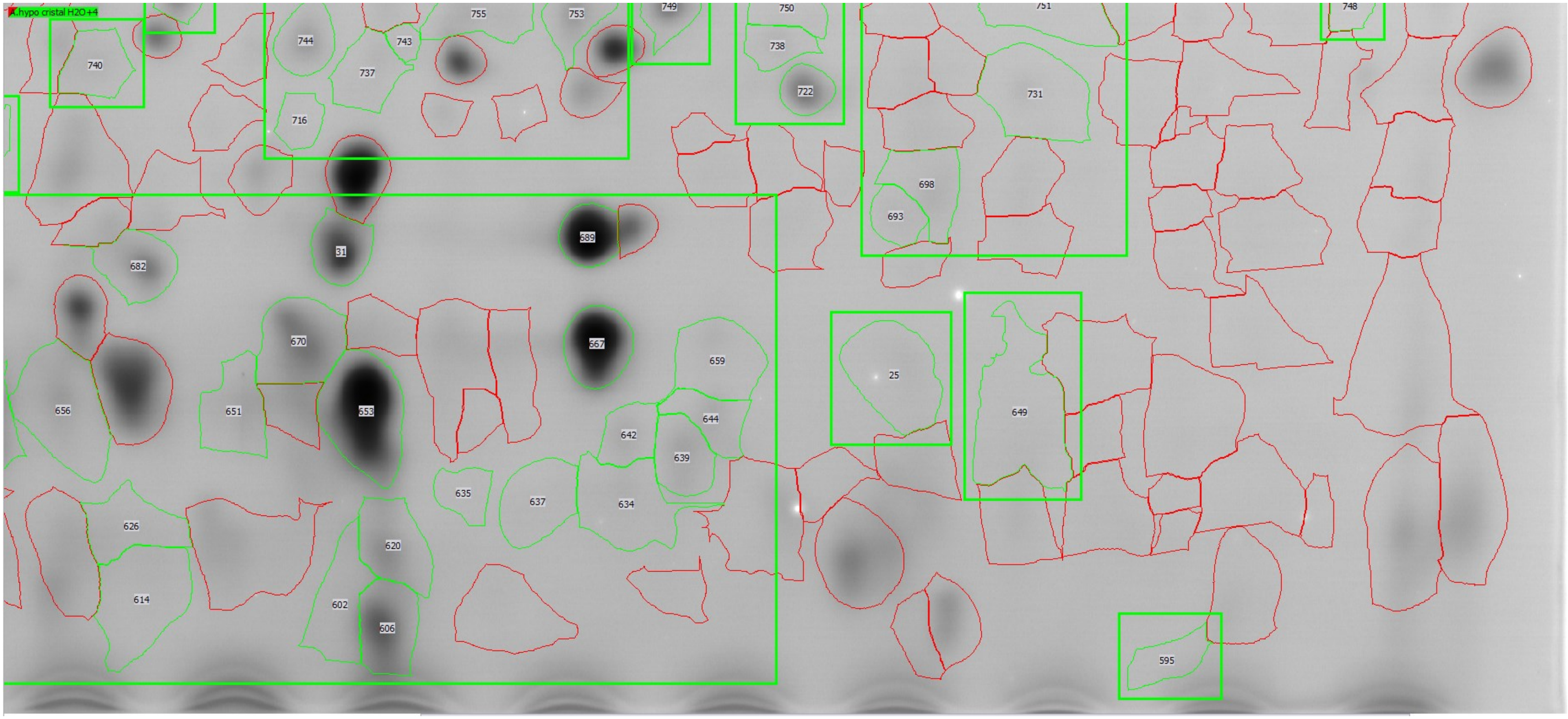

Hydrophobic Fraction (H0) HR-2DE

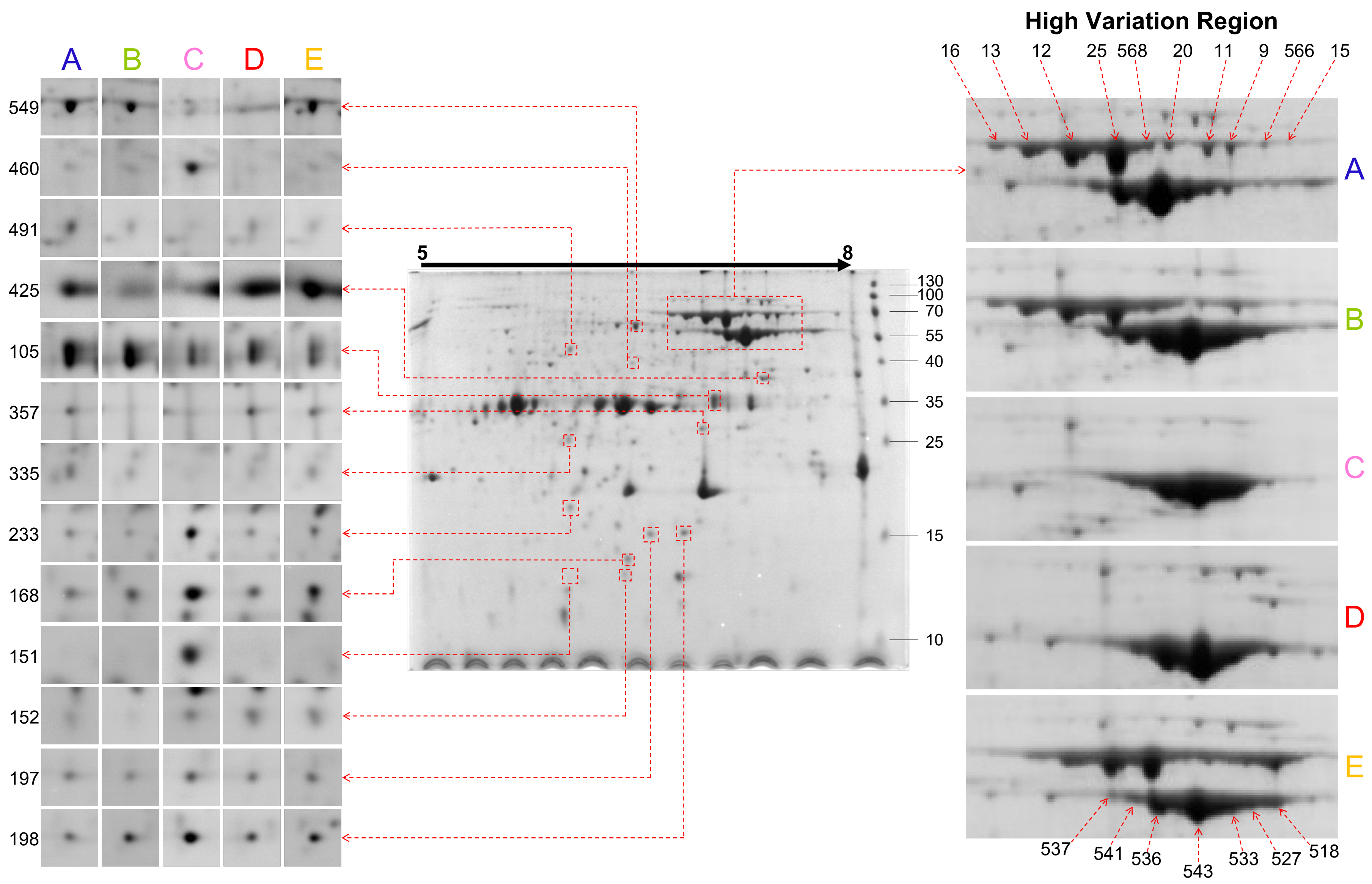

H0 HR-2DE Spot numbers

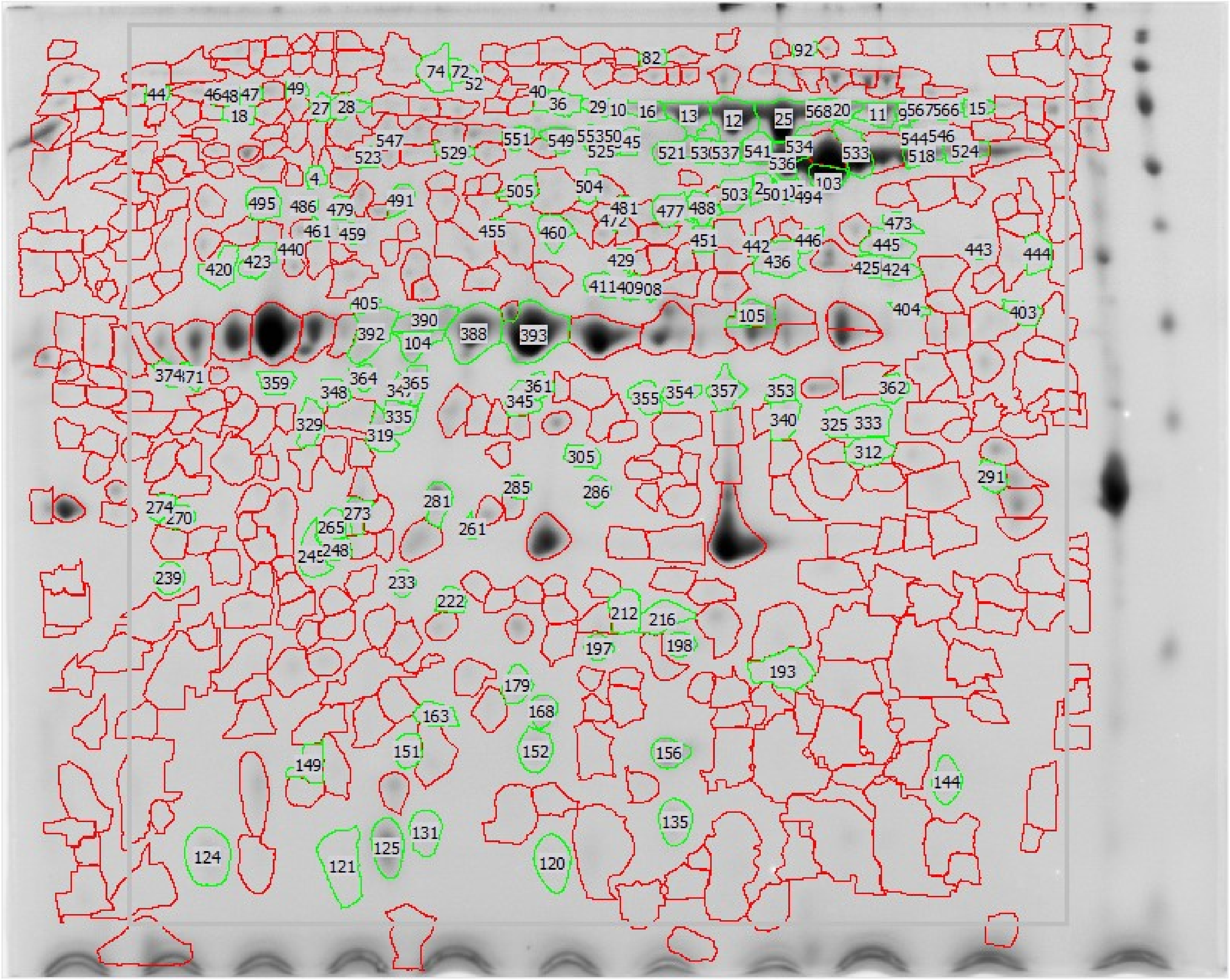

H0 HR-2DE Spot numbers Zoom 01

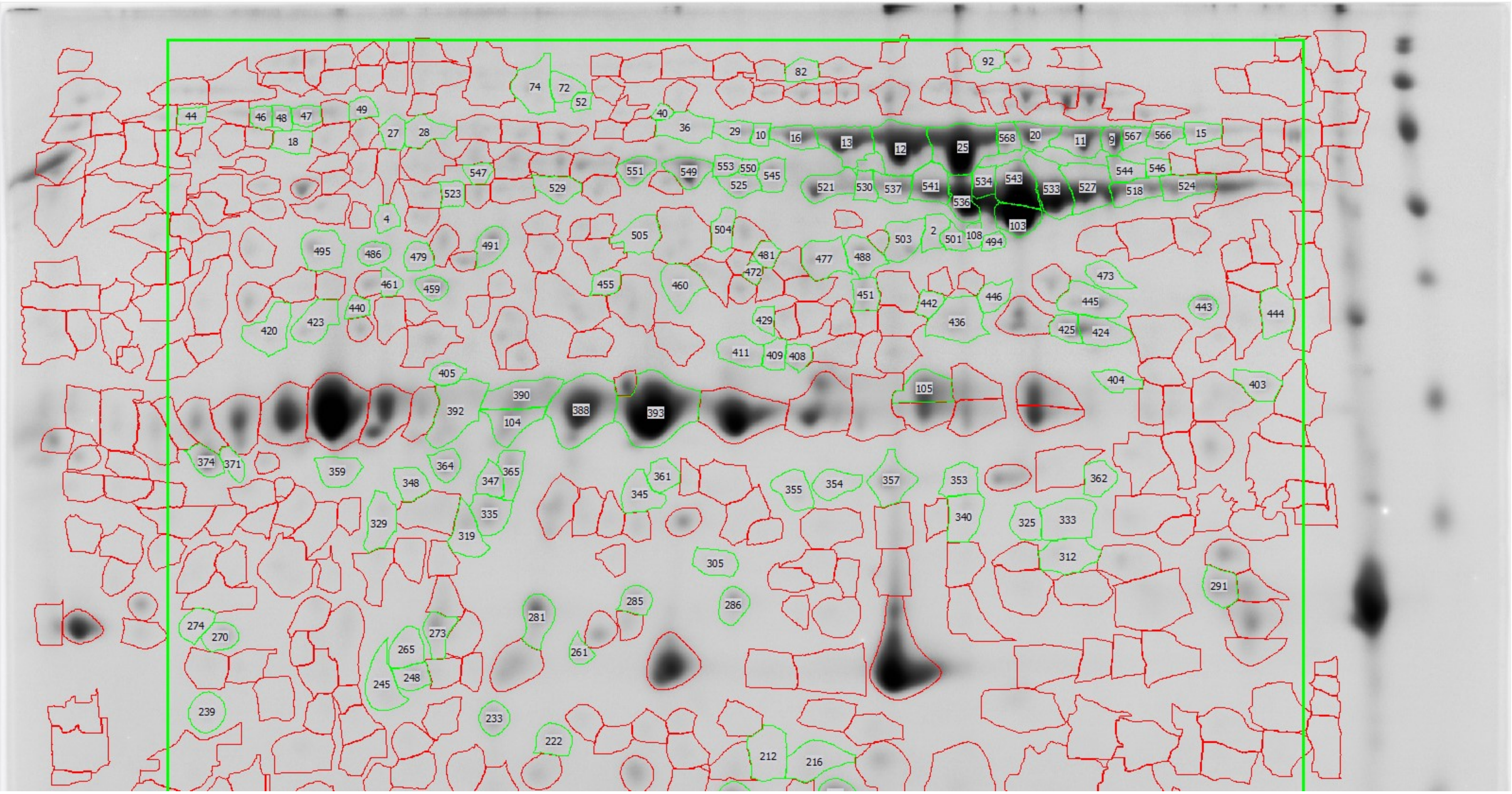

H0 HR-2DE Spot numbers Zoom 02

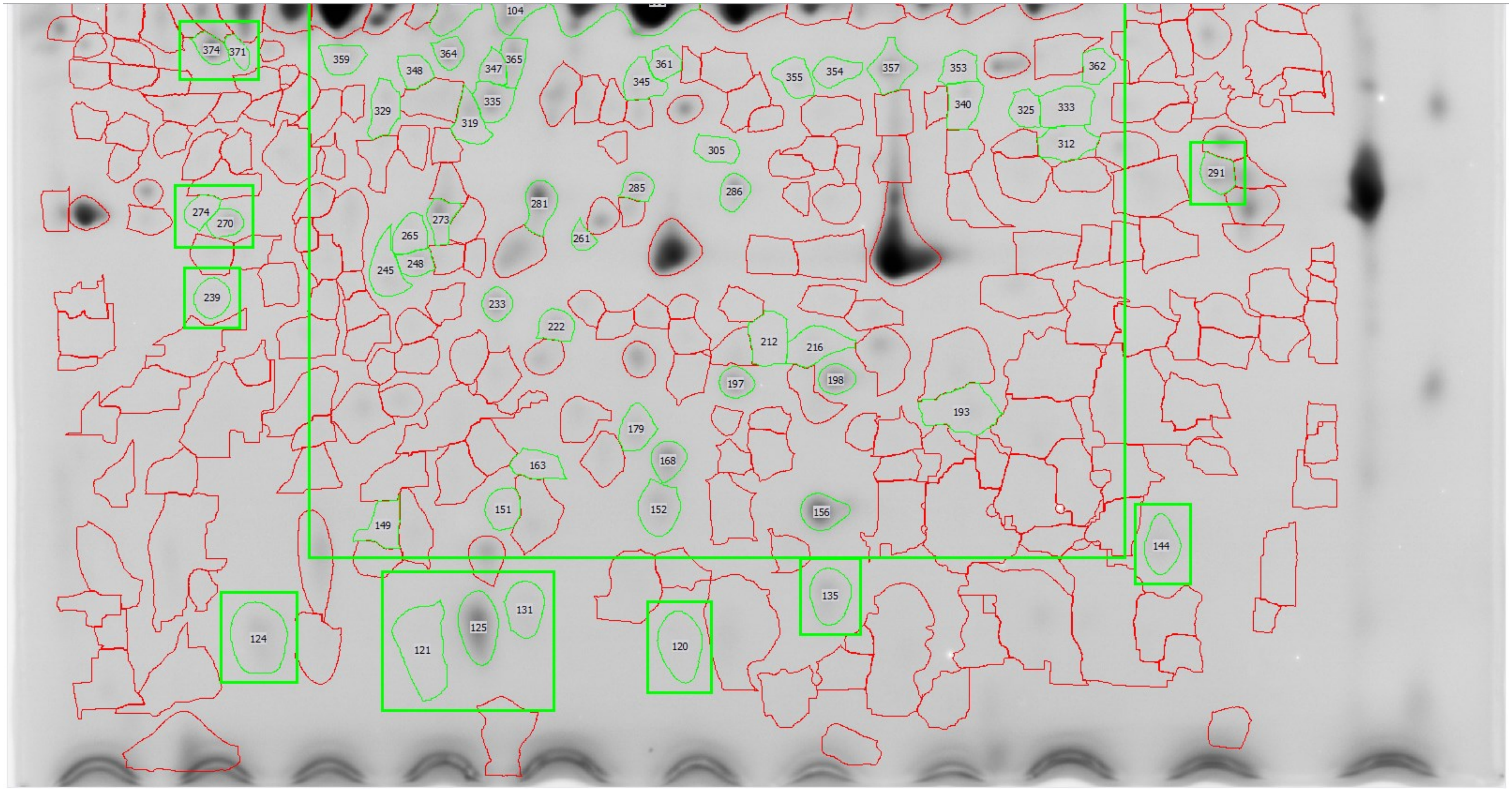

Supplement: Supplementary file 1 [file plants-13-02728-s001.zip › Supplementary Images_Spots numbers in gels.pdf]
