# Supplementary material for: Comparative Proteomic Analysis of Wild and Cultivated Amaranth Species Seeds by 2-DE and ESI-MS/MS
Source: Plants (Basel). 2024 Sep 29;13(19):2728. doi: 10.3390/plants13192728 (PMC11478449; doi:10.3390/plants13192728)
Supplement: Supplementary file 1 [file plants-13-02728-s001.zip › Supplementary Table S1.pdf]

Supplementary Table S1. Hydrophilic amaranth seed proteins identified by nLC-MS/MS in differentially 2-DE accumulated spots.

Spot No.[a]	Protein	Accession No.[b]	Ortholog [c]	Mr(kDa)/*pI* Exp.[d]	Mr(kDa)/*pI* Theo.[e]	Mascot Score[f]	PM/SC (%)[g]	emPAI[h]	Spot accumulation change[i] A B C D E	Metabolic Process[j]
28	Histone H4	AHYPO_005348-RA	H4_SOYBN	12.8/5.1	11.4/11.5	176	5/50	3.37	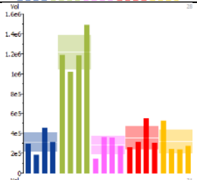	Genic regulation
31	Vicilin-like seed storage protein	AHYPO_018839-RA	AMP22_MACIN	13.4/6.4	60.9/6.6	124	4/8	0.27	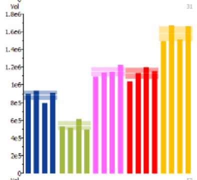	Seed maturation/ nutrient reservoir activity
53	Glutathione S-transferase	AHYPO_021773-RA	GSTF_SILVU	23.3/7.4	23.9/6.7	527	9/54	2.64	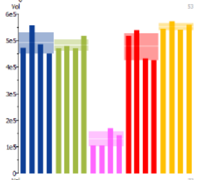	Reactive Oxygen Species (ROS) scavenging
72	18.3 kDa class I heat shock protein	AHYPO_013876-RA	HSP11_OXYRB	24.6/7.6	17.9/5.8	126	2/17	-	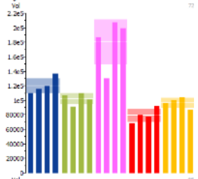	Stress response
95	Vicilin-like seed storage protein	AHYPO_006304-RA	VCL22_ARATH	25.3/5.2	61.9/5.9	524	6/12	0.46	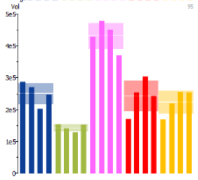	Seed maturation/ nutrient reservoir activity
103	Late embryogenesis abundant protein 31	AHYPO_006906-RA	LEA31_ARATH	27.6/5.4	28.6/5.0	273	4/18	0.64		Stress response

	Vicilin-like seed storage protein	AHYPO_006304-RA	VCL22_ARATH		61.9/5.9	188	4/9	0.26	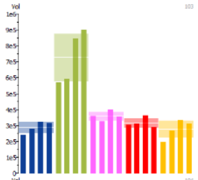	
104	Vicilin-like seed storage protein	AHYPO_006304-RA	VCL22_ARATH	28.1/7.4	61.9/5.9	271	3/8	0.19	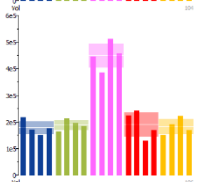	
106	Oil body-associated protein 2A	AHYPO_004342-RA	OBP2A_ARATH	28.8/6.9	25.5/7.1	439	7/27	1.75	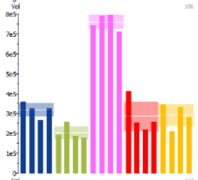	Lipid storage
112	Oil body-associated protein 1A	AHYPO_009953-RA	OBP1A_ARATH	29.5/6.7	26.6/6.2	477	6/30	1.89	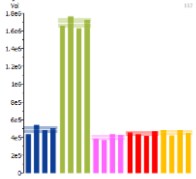	Lipid storage
125	Vicilin-like seed storage protein At2g18540	AHYPO_010140-RA	VCL21_ARATH	29.8/6.0	67.2/5.4	246	4/3	0.26	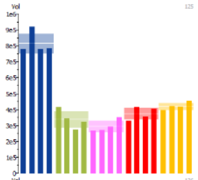	Seed maturation/ nutrient reservoir activity
126	Vicilin-like seed storage protein	AHYPO_006304-RA	VCL22_ARATH	30.2/7.8	61.9/5.9	1177	6/13	0.42	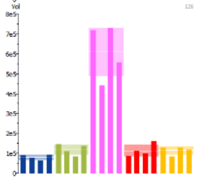	Seed maturation/ nutrient reservoir activity
130	Late embryogenesis abundant protein 31	AHYPO_006906-RA	LEA31_ARATH	30.0/5.1	28.6/5.0	782	9/42	2.06		Stress response

	Vicilin-like seed storage protein	AHYPO_006304-RA	VCL22_ARATH		61.9/5.9	229	4/7	0.26	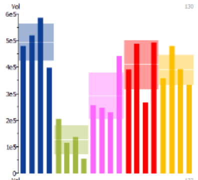	
	40S ribosomal protein S3-1	AHYPO_002178-RA	RS31_ARATH		26.8/9.6	128	2/12	0.30		
133	Vicilin-like seed storage protein	AHYPO_006304-RA	VCL22_ARATH	31.0/7.4	61.9/5.9	254	5/13	0.34	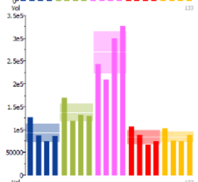	Seed maturation/ nutrient reservoir activity
137	Vicilin-like seed storage protein	AHYPO_006304-RA	VCL22_ARATH	32.1/5.8	61.9/5.9	710	9/19	0.67	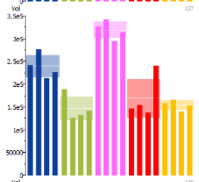	Seed maturation/ nutrient reservoir activity
138	Thiamine thiazole synthase 2, chloroplastic	AHYPO_004627-RA	THI42_VITVI	32.0/5.5	36.7/5.1	324	5/21	0.79	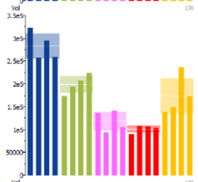	
	11S globulin	AHYPO_021282-RA	13SB_FAGES		77.6/7.0	202	5/8	0.26		
140	11S globulin	AHYPO_021282-RA	13SB_FAGES	32.1/5.7	77.6/7.0	239	9/15	0.56	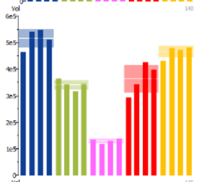	Seed maturation/ nutrient reservoir activity
145	Glucose and ribitol dehydrogenase	AHYPO_010964-RA	GRDH_DAUCA	33.5/7.0	31.5/6.5	819	11/33	2.29	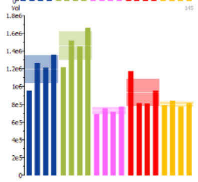	Glycolysis-TCA

151	11S globulin	AHYPO_001411-RA	CRU1_RAPSA	34.1/6.0	55.4/6.3	94	3/5	0.23	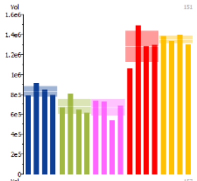	Seed maturation/ nutrient reservoir activity
157	11S globulin	AHYPO_001411-RA	CRU1_RAPSA	35.2/6.2	55.4/6.3	890	6/15	0.47	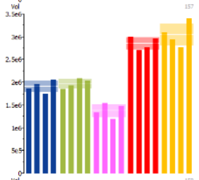	Seed maturation/ nutrient reservoir activity
	Agglutinin	AHYPO_007409-RA	Q38719_AMAHP		30.1/6.5	80	2/9	0.26		
159	Vicilin-like seed storage protein At2g18540	AHYPO_010140-RA	VCL21_ARATH	35.0/5.8	67.2/5.4	372	5/7	0.29	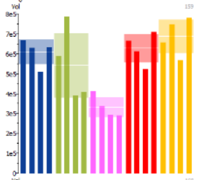	Seed maturation/ nutrient reservoir activity
169	14-3-3-like protein B	AHYPO_001919-RA	1433B_VICFA	35.3/5.1	29.7/4.9	172	3/18	0.45	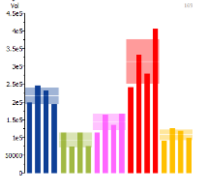	Signal transduction
175	Agglutinin	AHYPO_007409-RA	Q38719_AMAHP	35.8/6.8	30.1/6.5	559	5/27	0.83	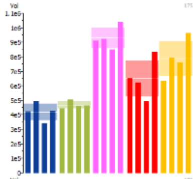	Carbohydrate binding
	Glucose and ribitol dehydrogenase	AHYPO_010964-RA	GRDH_DAUCA		31.5/6.5	267	5/18	0.78		
176	Agglutinin	AHYPO_007409-RA	Q38719_AMAHP	35.3/7.1	30.1/6.5	828	6/30	1.06	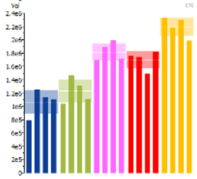	Carbohydrate binding
	Glucose and ribitol dehydrogenase	AHYPO_010964-RA	GRDH_DAUCA		31.5/6.5	358	6/21	1.00		

179	Agglutinin	AHYPO_007409-RA	Q38719_AMAHP	36.1/6.6	30.1/6.5	181	4/21	0.62	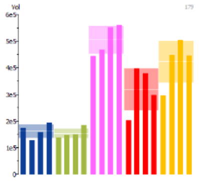	Carbohydrate binding
189	Glutelin type-D 1	AHYPO_000876-RA	GLUD1_ORYSJ	37.5/6.4	38.7/5.4	427	5/17	0.64	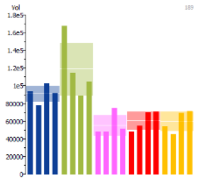	Seed maturation/ nutrient reservoir activity
193	Vicilin-like seed storage protein	AHYPO_018839-RA	AMP22_MACIN	37.3/6.9	60.9/6.6	99	4/8	0.30	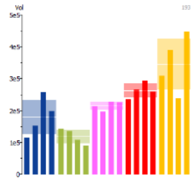	Seed maturation/ nutrient reservoir activity
194	Malate dehydrogenase 1, mitochondrial	AHYPO_012920-RA	MDHM1_ARATH	37.1/6.7	36.1/8.5	569	5/18	0.84	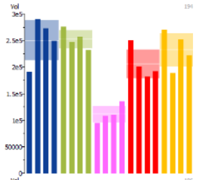	Glycolysis-TCA
	Agglutinin	AHYPO_007409-RA	Q38719_AMAHP		30.1/6.5	298	4/21	0.79		
196	Malate dehydrogenase, mitochondrial	AHYPO_004479-RA	MDHM_CITLA	37.7/6.4	36.2/8.4	110	2/8	0.24	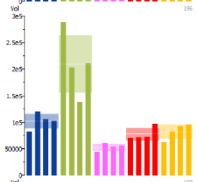	Glycolysis-TCA
197	Agglutinin	AHYPO_007409-RA	Q38719_AMAHP	37.3/6.6	30.1/6.5	270	4/21	0.60	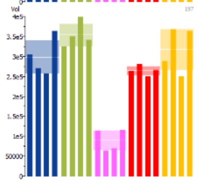	Carbohydrate binding

198	Agglutinin	AHYPO_007409-RA	Q38719_AMAHP	37.3/6.7	30.1/6.5	237	3/14	0.46	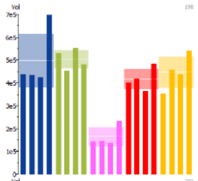	Carbohydrate binding
202	Omega-amidase, chloroplastic	AHYPO_006839-RA	NILP3_ARATH	37.6/6.5	40.4/8.6	106	2/8	0.21	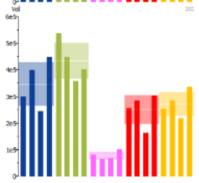	Amino acids metabolism
	Agglutinin	AHYPO_007409-RA	Q38719_AMAHP		30.1/6.5	96	2/9	0.28		
213	Vicilin-like seed storage protein	AHYPO_018839-RA	AMP22_MACIN	37.8/7.3	60.9/6.6	2433	10/20	0.90	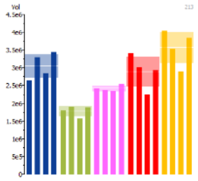	Seed maturation/ nutrient reservoir activity
218	Vicilin-like seed storage protein	AHYPO_018839-RA	AMP22_MACIN	37.7/5.2	60.9/6.6	602	9/18	0.69	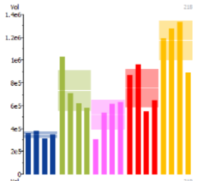	Seed maturation/ nutrient reservoir activity
	40S ribosomal protein SA	AHYPO_011970-RA	RSSA_SOYBN		30.1/5.0	362	3/11	0.42		
219	Vicilin-like seed storage protein	AHYPO_018839-RA	AMP22_MACIN	38.1/6.8	60.9/6.6	254	5/10	0.38	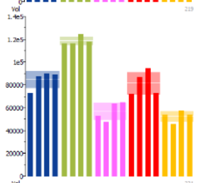	Seed maturation/ nutrient reservoir activity
	Annexin D2	AHYPO_020669-RA	ANXD2_ARATH		36.0/6.1	235	6/22	0.92		
	Malate dehydrogenase, mitochondrial	AHYPO_004479-RA	MDHM_CITLA		36.2/8.4	165	4/15	0.54		
221	Vicilin-like seed storage protein	AHYPO_018839-RA	AMP22_MACIN	38.0/5.4	60.9/6.6	367	7/15	0.50	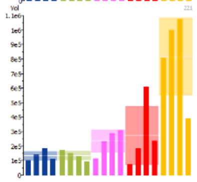	Seed maturation/ nutrient reservoir activity

231	Vicilin-like seed storage protein	AHYPO_018839-RA	AMP22_MACIN	38.2/7.0	60.9/6.6	669	10/20	0.79	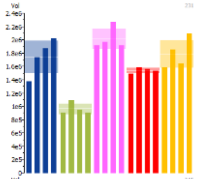	Seed maturation/ nutrient reservoir activity
245	Glutelin type-D 1	AHYPO_000876-RA	GLUD1_ORYSJ	39.6/6.0	38.7/5.4	224	5/17	0.63	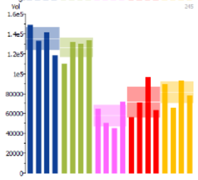	Seed maturation/ nutrient reservoir activity
254	Probable protein disulfide-isomerase A6	AHYPO_018548-RA	PDIA6_MEDSA	39.8/5.7	29.5/6.7	148	3/12	0.46	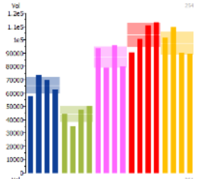	Protein folding
	Alpha-galactosidase	AHYPO_000222-RA	AGAL_COFAR		66.9/9.1	98	4/7	0.25		
	Caffeic acid 3-O-methyltransferase	AHYPO_000613-RA	COMT1_PRUDU		40.7/5.4	82	2/6	0.20		
261	11S globulin	AHYPO_021282-RA	13SB_FAGES	40.0/6.4	77.6/7.0	176	5/9	0.26	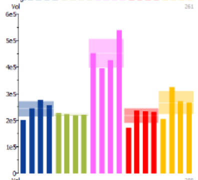	Seed maturation/ nutrient reservoir activity
	Beta-galactosidase 8	AHYPO_006310-RA	BGAL8_ARATH		87.5/6.4	171	3/5	0.13		
	Alcohol dehydrogenase 1	AHYPO_005892-RA	ADH1_PETHY		38.2/6.2	70	2/5	0.21		
288	11S globulin	AHYPO_021282-RA	13SB_FAGES	44.3/6.7	77.6/7.0	363	8/12	0.46	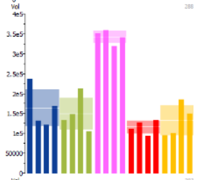	
	SNF1-related protein kinase regulatory subunit gamma-like PV42b	AHYPO_005043-RA	PV42B_ARATH		41.6/6.8	100	2/7	0.19		
	Vignain	AHYPO_000235-RA	CYSEP_RICCO		39.6/6.1	82	2/7	0.20		
292	Isocitrate dehydrogenase [NADP]	AHYPO_001339-RA	IDHC_TOBAC	45.3/6.4	46.2/5.8	113	5/12	0.53	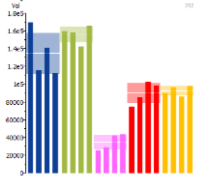	Glycolysis-TCA

301	UDP-D-apiose/UDP-D-xylose synthase 2	AHYPO_014290-RA	AXS2_ARATH	48.2/6.2	43.7/5.7	110	3/11	0.29	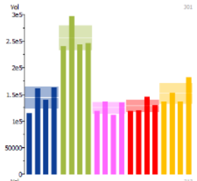	Cell wall-related
313	Vignain	AHYPO_000235-RA	CYSEP_RICCO	48.6/6.7	39.6/6.1	160	3/11	0.34	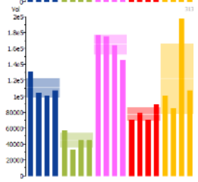	Proteolisis
316	11S globulin	AHYPO_021282-RA	13SB_FAGES	49.4/6.9	77.6/7.0	153	4/8	0.22	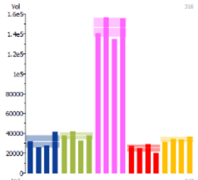	Seed maturation/ nutrient reservoir activity
347	11S globulin	AHYPO_021282-RA	13SB_FAGES	55.7/5.1	77.6/7.0	1046	9/17	0.59	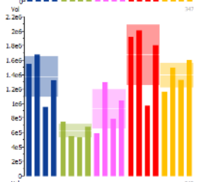	
	Agglutinin	AHYPO_007409-RA	Q38719_AMAHP		30.1/6.5	186	2/11	0.30		
	Vicilin-like seed storage protein	AHYPO_018839-RA	AMP22_MACIN		60.9/6.6	146	5/12	0.39		
348	Enolase	AHYPO_001182-RA	ENO_MESCR	58.4/5.9	48.2/5.5	1032	9/28	1.06	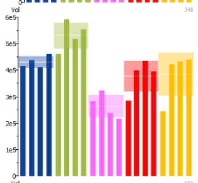	Glycolysis-TCA
352	Vicilin-like seed storage protein	AHYPO_018839-RA	AMP22_MACIN	56.3/5.1	60.9/6.6	843	12/25	1.07	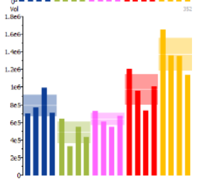	Seed maturation/ nutrient reservoir activity
	11S globulin	AHYPO_021282-RA	13SB_FAGES		77.6/7.0	275	5/9	0.27		

355	Vicilin-like seed storage protein	AHYPO_018839-RA	AMP22_MACIN	56.6/5.2	60.9/6.6	973	12/27	1.03	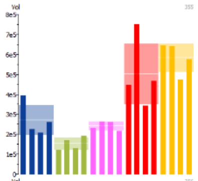	Seed maturation/ nutrient reservoir activity
	11S globulin	AHYPO_021282-RA	13SB_FAGES		77.6/7.0	95	3/6	0.15		
356	Vicilin-like seed storage protein	AHYPO_018839-RA	AMP22_MACIN	56.9/5.2	60.9/6.6	1132	12/28	1.15	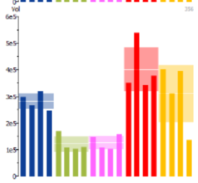	Seed maturation/ nutrient reservoir activity
362	Vicilin-like seed storage protein	AHYPO_018839-RA	AMP22_MACIN	57.3/5.3	60.9/6.6	231	6/14	0.43	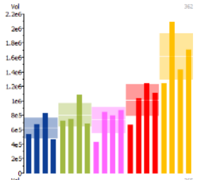	Seed maturation/ nutrient reservoir activity
	Elongation factor 1-alpha	AHYPO_001308-RA	EF1A_ORYSJ		50.6/9.0	162	3/12	0.24		
365	Catalase	AHYPO_007232-RA	CATA_IPOBA		52.3/6.8	82	3/6	0.23	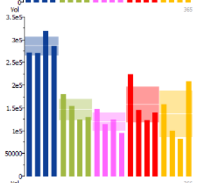	Seed maturation/ nutrient reservoir activity
366	Vicilin-like seed storage protein	AHYPO_018839-RA	AMP22_MACIN	59.1/7.3	60.9/6.6	329	4/9	0.29	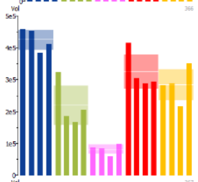	Seed maturation/ nutrient reservoir activity
367	Vicilin-like seed storage protein	AHYPO_018839-RA	AMP22_MACIN	58.3/5.4	60.9/6.6	696	11/24	0.91	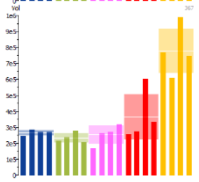	Seed maturation/ nutrient reservoir activity

370	11S globulin	AHYPO_021282-RA	13SB_FAGES	58.5/7.1	77.6/7.0	1082	10/19	0.61	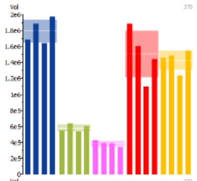	Seed maturation/ nutrient reservoir activity
	Vicilin-like seed storage protein	AHYPO_018839-RA	AMP22_MACIN		60.9/6.6	371	9/20	0.72		
372	11S globulin	AHYPO_021282-RA	13SB_FAGES	59.2/7.1	77.6/7.0	472	8/14	0.46	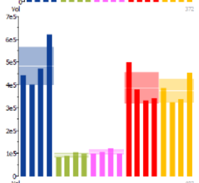	Seed maturation/ nutrient reservoir activity
	Vicilin-like seed storage protein	AHYPO_018839-RA	AMP22_MACIN		60.9/6.6	424	7/17	0.53		
403	Granule-bound starch synthase 1, chloroplastic/amyloplastic	AHYPO_011500-RA	SSG1_MANES	66.0/5.8	62.7/6.5	89	3/5	0.20	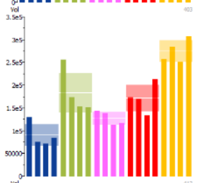	Starch biosynthetic process
413	Vicilin-like seed storage protein	AHYPO_018839-RA	AMP22_MACIN	67.9/5.4	60.9/6.6	155	6/14	0.47	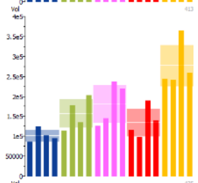	Seed maturation/ nutrient reservoir activity
425	2,3-bisphosphoglycerate-independent phosphoglycerate mutase	AHYPO_016738-RA	PMGI_MESCR	72.8/5.9	60.1/5.5	252	7/14	0.51	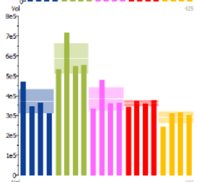	Glycolysis-TCA
	Pyruvate decarboxylase 1	AHYPO_019658-RA	PDC1_ARATH		44.9/5.6	104	2/6	0.17		
426	Granule-bound starch synthase 1, chloroplastic/amyloplastic	AHYPO_011500-RA	SSG1_MANES	70.0/6.8	62.7/6.5	570	13/27	1.24	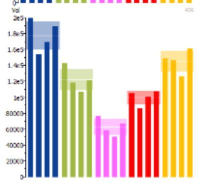	Starch biosynthetic process
	NADP-dependent malic enzyme	AHYPO_020870-RA	MAOX_VITVI		65.6/6.0	117	4/8	0.27		
427	Granule-bound starch synthase 1, chloroplastic/amyloplastic	AHYPO_011500-RA	SSG1_MANES	70.3/6.7	62.7/6.5	144	6/10	0.45		Starch biosynthetic process

	Vicilin-like seed storage protein	AHYPO_018839-RA	AMP22_MACIN		60.9/6.6	75	3/6	0.21	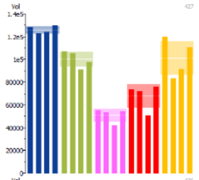	
436	NADP-dependent malic enzyme	AHYPO_020870-RA	MAOX_VITVI	72.5/6.5	65.6/6.0	273	10/13	0.48	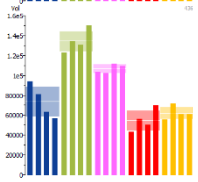	Glycolysis-TCA
444	5-methyltetrahydropteroyltriglutamate--homocysteine methyltransferase	AHYPO_017360-RA	METE_MESCR	82.8/6.4	84.6/6.1	299	5/7	0.26	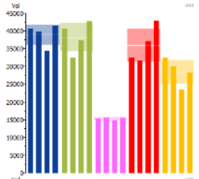	Amino acids metabolism
449	Heat shock 70 kDa protein, mitochondrial	AHYPO_010311-RA	HSP7M_PHAVU	79/5.5	33.3/5.0	149	4/12	0.56	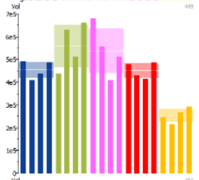	Stress response
454	Embryonic protein DC-8	AHYPO_000638-RA	LEAD8_DAUCA	79.0/7.6	65.1/6.6	90	2/3	0.13	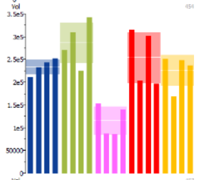	Stress response
457	Embryonic protein DC-8	AHYPO_000638-RA	LEAD8_DAUCA	85.7/6.5	65.1/6.6	238	6/10	0.43	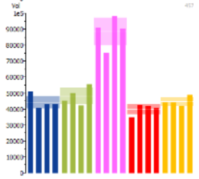	Stress response
	Agglutinin	AHYPO_007409-RA	Q38719_AMAHP		30.1/6.5	70	2/9	0.29		

466	Seed biotin-containing protein SBP65	AHYPO_013747-RA	SBP65_PEA	90.6/7.3	72.4/6.7	84	2/5	0.11	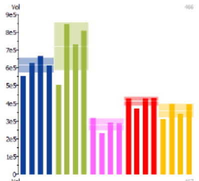	Stress response
467	Seed biotin-containing protein SBP65	AHYPO_013747-RA	SBP65_PEA	89.1/7.3	72.4/6.7	96	3/5	0.17	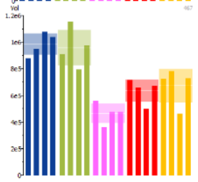	Stress response
472	5-methyltetrahydropteroyltriglutamate--homocysteine methyltransferase	AHYPO_017357-RA	METE_MESCR	94.2/6.7	84.7/6.1	396	7/10	0.38		Amino acids metabolism
	5-methyltetrahydropteroyltriglutamate--homocysteine methyltransferase	AHYPO_017360-RA	METE_MESCR		84.6/6.1	365	7/10	0.38	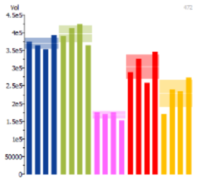	
	5-methyltetrahydropteroyltriglutamate--homocysteine methyltransferase	AHYPO_022179-RA	METE_CATRO		89.4/6.3	248	4/5	0.19		
478	5-methyltetrahydropteroyltriglutamate--homocysteine methyltransferase	AHYPO_017360-RA	METE_MESCR	95.8/6.6	84.6/6.1	953	19/30	1.24		Amino acids metabolism
	5-methyltetrahydropteroyltriglutamate--homocysteine methyltransferase	AHYPO_017357-RA	METE_MESCR		84.7/6.1	885	18/28	1.15	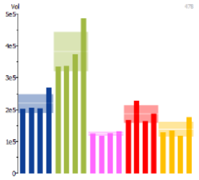	
	5-methyltetrahydropteroyltriglutamate--homocysteine methyltransferase	AHYPO_022179-RA	METE_CATRO		89.4/6.3	291	6/8	0.27		
479	5-methyltetrahydropteroyltrig	AHYPO_017360-RA	METE_MESCR	97.5/6.5	84.6/6.1	547	11/18	0.59		Amino acids metabolism

	lutamate--homocysteine methyltransferase							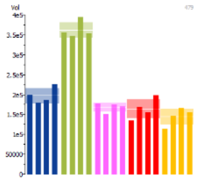	
	5-methyltetrahydropteroyltriglutamate--homocysteine methyltransferase	AHYPO_017357-RA	METE_MESCR		84.7/6.1	539	11/18	0.59	
	Glycine--tRNA ligase, mitochondrial 1	AHYPO_003493-RA	SYGM1_ARATH		76.0/5.9	302	7/11	0.39	
480	5-methyltetrahydropteroyltriglutamate--homocysteine methyltransferase	AHYPO_017360-RA	METE_MESCR	99.6/6.4	84.6/6.1	517	8/12	0.45	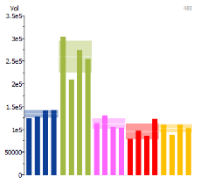 Amino acids metabolism
	5-methyltetrahydropteroyltriglutamate--homocysteine methyltransferase	AHYPO_017357-RA	METE_MESCR		84.7/6.1	517	8/12	0.45	
483	Superoxide dismutase [Fe] 2, chloroplastic	AHYPO_009159-RA	SODF2_ARATH	23.5/5.6	28.3/6.3	153	5/30	0.87	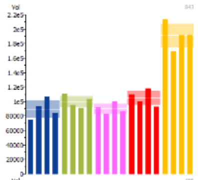 Reactive Oxygen Species (ROS) scavenging
	Vicilin-like seed storage protein	AHYPO_018839-RA	AMP22_MACIN		60.9/6.6	122	3/6	0.19	
488	Seed biotin-containing protein SBP65	AHYPO_013747-RA	SBP65_PEA	105.3/5.1	72.4/6.7	70	2/3	0.11	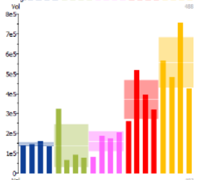
492	Poly [ADP-ribose] polymerase 3	AHYPO_003773-RA	PARP3_SOYBN	109.9/5.9	81.9/5.2	529	10/18	0.69	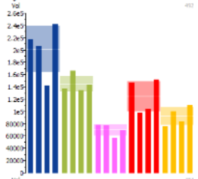 Reactive Oxygen Species (ROS) scavenging
494	5-methyltetrahydropteroyltriglutamate--homocysteine methyltransferase	AHYPO_017360-RA	METE_MESCR	109.5/5.4	84.6/6.1	94	3/4	0.13	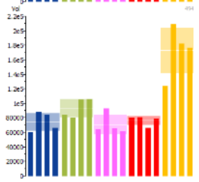 Amino acids metabolism

497	Poly [ADP-ribose] polymerase 3	AHYPO_003773-RA	PARP3_SOYBN	106.2/5.4	81.9/5.2	259	7/9	0.45	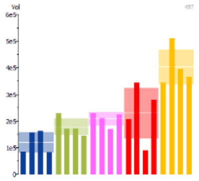	Reactive Oxygen Species (ROS) scavenging
503	5-methyltetrahydropteroyltriglutamate--homocysteine methyltransferase	AHYPO_017360-RA	METE_MESCR	112.3/6.0	84.6/6.1	145	5/7	0.26	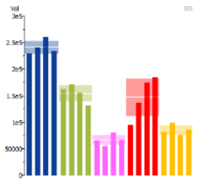	Amino acids metabolism
	5-methyltetrahydropteroyltriglutamate--homocysteine methyltransferase	AHYPO_017357-RA	METE_MESCR		84.7/6.1	129	5/7	0.26		
	Poly [ADP-ribose] polymerase 3	AHYPO_003773-RA	PARP3_SOYBN		81.9/5.2	112	4/6	0.21		
506	Elongation factor 2	AHYPO_001926-RA	EF2_BETVU	115.3/6.5	93.9/5.9	240	5/7	0.23	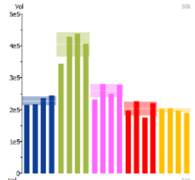	Translation
516	Chaperone protein ClpB1	AHYPO_008070-RA	CLPB1_ARATH	116.8/6.4	100.7/5.7	585	15/21	0.72	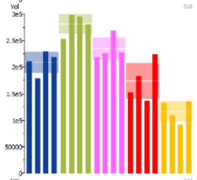	Stress response
	Chaperone protein ClpB1	AHYPO_012598-RA	CLPB1_ARATH		81.4/6.4	248	8/13	0.43		
	Low-temperature-induced 65 kDa protein	AHYPO_018897-RA	LTI65_ARATH		87.0/5.7	162	3/4	0.13		
518	Poly [ADP-ribose] polymerase 3	AHYPO_003773-RA	PARP3_SOYBN	125.1/5.4	81.9/5.2	248	7/10	0.40	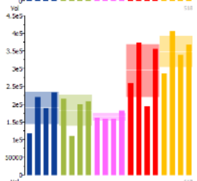	Reactive Oxygen Species (ROS) scavenging
	Cell division cycle protein 48 homolog	AHYPO_012429-RA	CDC48_SOYBN		90.7/5.2	131	4/5	0.19		
	Heat shock protein 83	AHYPO_010680-RA	HSP83_IPONI		39.9/4.5	115	2/7	0.21		
617	11S globulin	AHYPO_021282-RA	13SB_FAGES	9.5/5.4	77.6/7.0	179	4/6	0.21	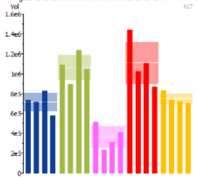	

670	Seed maturation protein	AHYPO_021176-RA	A0A072VAH4_MEDTR	12.3/6.3	10.4/6.1	147	2/26	0.90	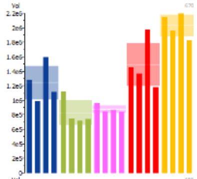	Stress response
682	Vicilin-like seed storage protein	AHYPO_018839-RA	AMP22_MACIN	13.2/6.0	60.9/6.6	1220	4/8	0.27	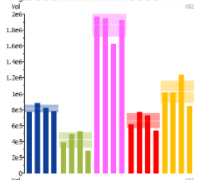	Seed maturation/ nutrient reservoir activity
683	Vicilin-like seed storage protein	AHYPO_018839-RA	AMP22_MACIN	13.0/5.7	60.9/6.6	188	3/8	0.21	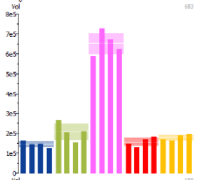	Seed maturation/ nutrient reservoir activity
689	Vicilin-like seed storage protein	AHYPO_018839-RA	AMP22_MACIN	13.3/6.7	60.9/6.6	1091	3/8	0.19	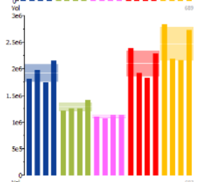	Seed maturation/ nutrient reservoir activity
692	Vicilin-like seed storage protein	AHYPO_018839-RA	AMP22_MACIN	13.7/6.0	60.9/6.6	474	3/8	0.19	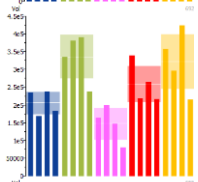	Seed maturation/ nutrient reservoir activity
693	Vicilin-like seed storage protein	AHYPO_018839-RA	AMP22_MACIN	13.5/7.2	60.9/6.6	304	2/5	0.13	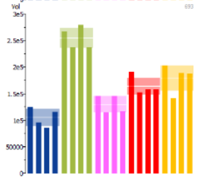	Seed maturation/ nutrient reservoir activity

694	Vicilin-like seed storage protein	AHYPO_018839-RA	AMP22_MACIN	13.6/5.8	60.9/6.6	221	2/5	0.13	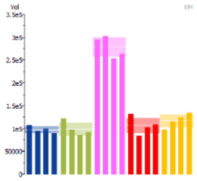	Seed maturation/ nutrient reservoir activity
696	Major allergen Mal d 1	AHYPO_006247-RA	MAL12_MALDO	13.7/5.7	17.9/6.2	210	6/46	2.32	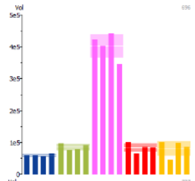	Defense response
	Vicilin-like seed storage protein	AHYPO_018839-RA	AMP22_MACIN		60.9/6.6	168	2/5	0.13		
737	Major allergen Mal d 1	AHYPO_006247-RA	MAL12_MALDO	15.9/6.4	17.9/6.2	301	5/38	1.63	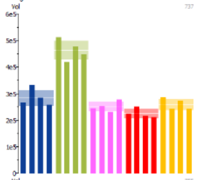	Defense response
	Dessication-induced 1VOC superfamily protein	AHYPO_014874-RA	Q9LQP1_ARATH		11.0/5.0	167	2/20	0.85		
755	Vicilin-like seed storage protein	AHYPO_018839-RA	AMP22_MACIN	17.3/6.6	60.9/6.6	419	3/8	0.21	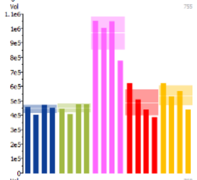	Seed maturation/ nutrient reservoir activity
760	Peroxiredoxin-2B	AHYPO_015627-RA	PRX2B_ARATH	17.4/6.1	17.5/5.9	564	9/39	4.62	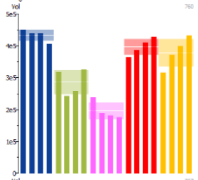	Reactive Oxygen Species (ROS) scavenging
767	CBS domain-containing protein CBSX3, mitochondrial	AHYPO_009852-RA	CBSX3_ARATH	18.1/6.6	22.4/7.9	148	5/18	1.28	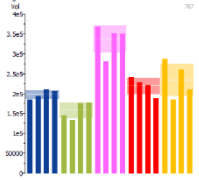	Reactive Oxygen Species (ROS) scavenging

771	18.3 kDa class I heat shock protein	AHYPO_013876-RA	HSP11_OXYRB	17.7/6.0	17.9/5.8	547	6/43	3.24	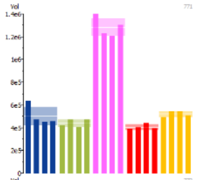	Stress response
772	18.3 kDa class I heat shock protein	AHYPO_013876-RA	HSP11_OXYRB	18.1/5.6	17.9/5.8	413	3/24	0.88	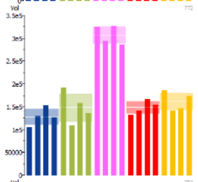	Stress response
784	11S globulin	AHYPO_021282-RA	13SB_FAGES	19.0/5.7	77.6/7.0	495	7/9	0.39	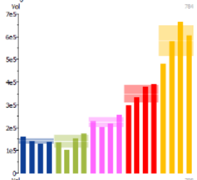	Seed maturation/ nutrient reservoir activity
798	11S globulin	AHYPO_021282-RA	13SB_FAGES	19.6/6.4	77.6/7.0	107	3/6	0.15	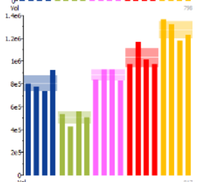	Seed maturation/ nutrient reservoir activity
817	Vicilin-like seed storage protein At2g18540	AHYPO_010140-RA	VCL21_ARATH	20.8/5.3	67.2/5.4	142	2/3	0.11	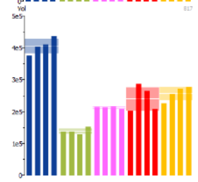	Seed maturation/ nutrient reservoir activity
821	Vicilin-like seed storage protein At2g18540	AHYPO_010140-RA	VCL21_ARATH	20.0/5.1	67.2/5.4	324	5/12	0.30	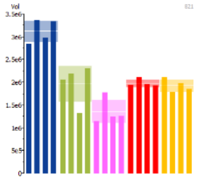	Seed maturation/ nutrient reservoir activity

[a]Spot number assigned by Melanie software. [b]Accession number according to the database reported by Clouse et al. 2016. [c]UniProtKB/Swiss-Prot ortholog identifier assigned by Trinotate. [d]Experimental mass and isoelectric point. [e]Theoretical mass and isoelectric point. [f]MASCOT Score, individual ion scores statistically significant at $p<0.001$, only identifications with peptide matches above the identity threshold when FDR≤1% were considered true. [g]Peptides Matched/Sequence Coverage. [h]Exponentially Modified Protein Abundance Index. [i]Protein spot accumulation change histograms ($p\leq0.001$ and fold change≥2.0): A, *A. hybridus*; B, *A. powellii*; C, *A. cruentus* cv Amaranteca; D, *A. hypochondriacus* cv Opaca; E, *A. hypochondriacus* cv Cristalina. [j]The metabolic process of the main identified protein based on emPAI is shown.
