# Supplementary material for: Comparative Proteomic Analysis of Wild and Cultivated Amaranth Species Seeds by 2-DE and ESI-MS/MS
Source: Plants (Basel). 2024 Sep 29;13(19):2728. doi: 10.3390/plants13192728 (PMC11478449; doi:10.3390/plants13192728)
Supplement: Supplementary file 1 [file plants-13-02728-s001.zip › Supplementary Table S2.pdf]

Supplementary Table S2. Hydrophobic amaranth seed proteins identified by nLC-MS/MS in differentially 2-DE accumulated spots.

Spot No.[a]	Protein	Accession No.[b]	Ortholog [c]	Mr(kDa)/*pI* Exp.[d]	Mr(kDa)/*pI* Theo.[e]	Mascot Score[f]	PM/SC (%)[g]	emPAI[h]	Spot accumulation change[i] A B C D E	Metabolic Process[j]
2	11S globulin	AHYPO_021282-RA	13SB_FAGES	55.4/7.0	77.6/7.0	287	5/9	0.33	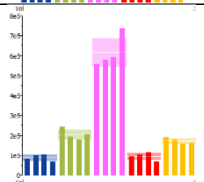	Seed maturation/ nutrient reservoir activity
9	Granule-bound starch synthase I, chloroplastic/amyloplastic	AHYPO_011500-RA	SSG1_MANES	73.6/7.4	62.7/6.5	988	19/42	2.23	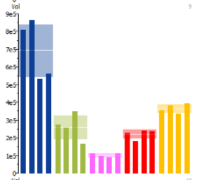	Starch biosynthetic process
	11S globulin	AHYPO_021282-RA	13SB_FAGES		77.6/7.0	549	10/18	0.69		
10	Granule-bound starch synthase I, chloroplastic/amyloplastic	AHYPO_011500-RA	SSG1_MANES	73.3/6.6	62.7/6.5	1096	18/38	2.38	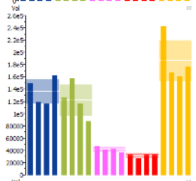	Starch biosynthetic process
11	Granule-bound starch synthase I, chloroplastic/amyloplastic	AHYPO_011500-RA	SSG1_MANES	74.2/7.3	62.7/6.5	1349	22/50	3.09	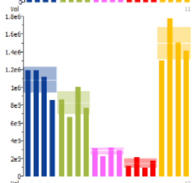	Starch biosynthetic process
	11S globulin	AHYPO_021282-RA	13SB_FAGES		77.6/7.0	180	4/6	0.21		
12	Granule-bound starch synthase I, chloroplastic/amyloplastic	AHYPO_011500-RA	SSG1_MANES	69.2/7.0	62.7/6.5	1775	20/43	2.98	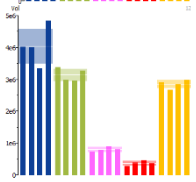	Starch biosynthetic process

13	Granule-bound starch synthase I, chloroplastic/amyloplastic	AHYPO_011500-RA	SSG1_MANES	70.8/6.8	62.7/6.5	1508	19/43	2.56	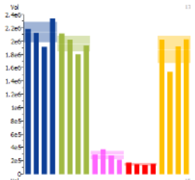	Starch biosynthetic process
15	Granule-bound starch synthase I, chloroplastic/amyloplastic	AHYPO_011500-RA	SSG1_MANES	71.9/7.5	62.7/6.5	1298	23/52	3.34	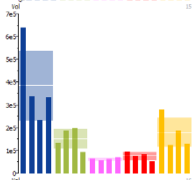	Starch biosynthetic process
	11S globulin	AHYPO_021282-RA	13SB_FAGES		77.6/7.0	331	6/9	0.33		
16	Granule-bound starch synthase I, chloroplastic/amyloplastic	AHYPO_011500-RA	SSG1_MANES	72.7/6.7	62.7/6.5	1666	22/45	3.41	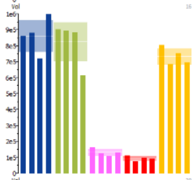	Starch biosynthetic process
20	Granule-bound starch synthase I, chloroplastic/amyloplastic	AHYPO_011500-RA	SSG1_MANES	75.0/7.2	62.7/6.5	1295	21/45	3.60	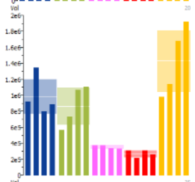	Starch biosynthetic process
25	Granule-bound starch synthase I, chloroplastic/amyloplastic	AHYPO_011500-RA	SSG1_MANES	68.7/7.0	62.7/6.5	1346	16/33	1.58	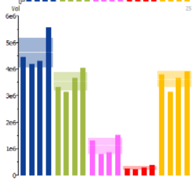	Starch biosynthetic process
27	Granule-bound starch synthase I, chloroplastic/amyloplastic	AHYPO_011500-RA	SSG1_MANES	73.8/5.7	62.7/6.5	852	10/25	0.74	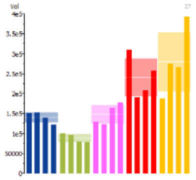	Starch biosynthetic process
	11S globulin	AHYPO_021282-RA	13SB_FAGES		77.6/7.0	687	12/22	0.71		
	Chaperonin CPN60, mitochondrial	AHYPO_003944-RA	CH60A_ARATH		57.7/6.4	462	12/25	1.05		

28	11S globulin	AHYPO_021282-RA	13SB_FAGES	75.3/5.7	77.6/7.0	597	13/22	0.84	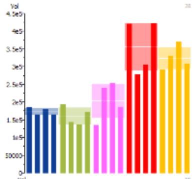	Seed maturation/ nutrient reservoir activity
	Granule-bound starch synthase I, chloroplastic/amyloplastic	AHYPO_011500-RA	SSG1_MANES		62.7/6.5	324	7/16	0.50		
29	Granule-bound starch synthase I, chloroplastic/amyloplastic	AHYPO_011500-RA	SSG1_MANES	74.7/6.5	62.7/6.5	1380	22/49	3.68	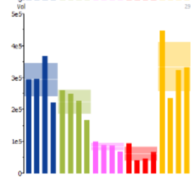	Starch biosynthetic process
30	11S globulin	AHYPO_021282-RA	13SB_FAGES	74.4/5.6	77.6/7.0	367	11/20	0.68	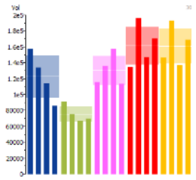	Seed maturation/ nutrient reservoir activity
	Granule-bound starch synthase I, chloroplastic/amyloplastic	AHYPO_011500-RA	SSG1_MANES		62.7/6.5	270	6/14	0.42		
	Chaperonin CPN60, mitochondrial	AHYPO_003944-RA	CH60A_ARATH		57.7/6.4	242	11/24	1.00		
	Chaperonin CPN60, mitochondrial	AHYPO_014580-RA	CH60A_ARATH			197	8/15	0.61		
36	Granule-bound starch synthase I, chloroplastic/amyloplastic	AHYPO_011500-RA	SSG1_MANES	77.3/6.4	62.7/6.5	1162	19/41	2.82	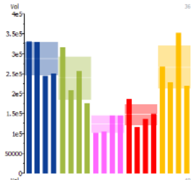	Starch biosynthetic process
40	Granule-bound starch synthase I, chloroplastic/amyloplastic	AHYPO_011500-RA	SSG1_MANES	85.5/6.3	62.7/6.5	132	3/8	0.20	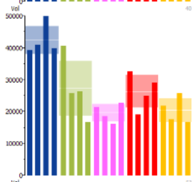	Starch biosynthetic process
52	NADH dehydrogenase [ubiquinone] iron-sulphur protein 1, mitochondrial	AHYPO_001360-RA	NDUS1_SOLTU	93.7/6.12	81.0/6.1	370	10/17	0.63	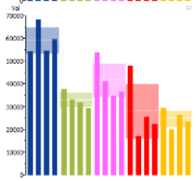	Proton transmembrane transport

72	Alpha-xylosidase 1	AHYPO_005558-RA	XYL1_ARATH	100.8/6.1	104.0/5.9	120	4/4	0.17	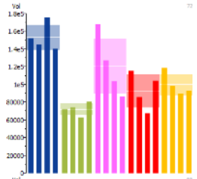	Cell wall-related
92	11S globulin	AHYPO_021282-RA	13SB_FAGES	124.3/7.1	77.6/7.0	158	5/9	0.27	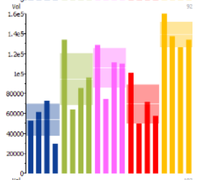	Seed maturation/ nutrient reservoir activity
103	11S globulin	AHYPO_021282-RA	13SB_FAGES	54.0/7.1	77.6/7.0	1121	10/18	0.59	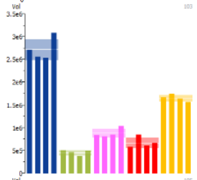	Seed maturation/ nutrient reservoir activity
105	Agglutinin	AHYPO_007409-RA	Q38719_AMAHP	37.0/7.0	30.1/6.5	354	6/30	1.24	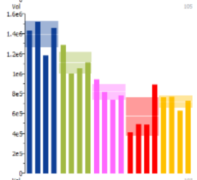	Carbohydrate binding
108	11S globulin	AHYPO_021282-RA	13SB_FAGES	54.0/7.1	77.6/7.0	251	6/11	0.33	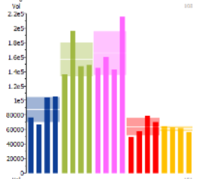	Seed maturation/ nutrient reservoir activity
151	Vicilin-like seed storage protein	AHYPO_018839-RA	AMP22_MACIN	13.0/5.9	60.9/6.6	183	2/5	0.13	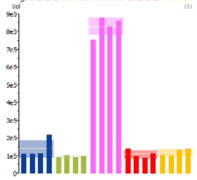	Seed maturation/ nutrient reservoir activity

152	Vicilin-like seed storage protein	AHYPO_018839-RA	AMP22_MACIN	13.1/6.3	60.9/6.6	975	3/8	0.20	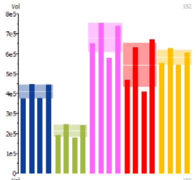	Seed maturation/ nutrient reservoir activity
156	Vicilin-like seed storage protein	AHYPO_018839-RA	AMP22_MACIN	13.4/6.7	60.9/6.6	1147	4/8	0.30	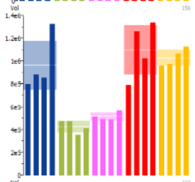	Seed maturation/ nutrient reservoir activity
163	Vicilin-like seed storage protein	AHYPO_018839-RA	AMP22_MACIN	13.8/6.0	60.9/6.6	116	2/5	0.14	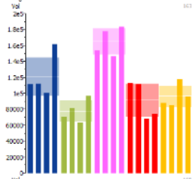	Seed maturation/ nutrient reservoir activity
168	Late embryogenesis abundant protein B19.3	AHYPO_008005-RA	LE193_HORVU	14.1/6.3	9.7/5.9	147	2/26	1.06	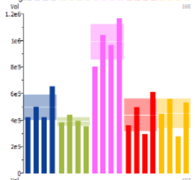	Stress response
	Protein SLE2	AHYPO_019862-RA	SLE2_SOYBN		8.5/5.9	62	2/30	1.25		
197	17.9 kDa class II heat shock protein	AHYPO_006834-RA	HSP21_SOYBN	15.8/6.5	18.2/6.2	222	4/30	1.23	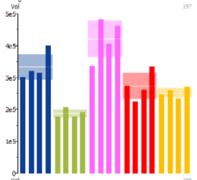	Stress response
198	17.4 kDa class I heat shock protein;	AHYPO_012223-RA	HSP17_ARATH	16.2/6.8	17.4/6.3	178	3/21	0.83	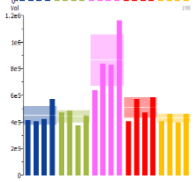	Stress response

233	18.3 kDa class I heat shock protein	AHYPO_013876-RA	HSP11_OXYRB	17.9/5.9	17.9/5.8	326	5/32	1.66	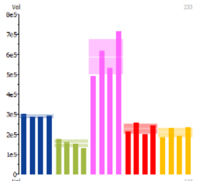	Stress response
248	17.4 kDa class I heat shock protein	AHYPO_013881-RA	HSP17_ARATH	19.4/5.7	19.1/5.6	170	4/25	1.55	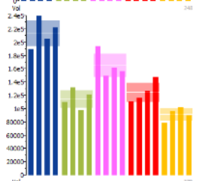	Stress response
270	Vicilin-like seed storage protein At2g18540	AHYPO_010140-RA	VCL21_ARATH	21.2/5.3	67.2/5.4	115	2/3	0.12	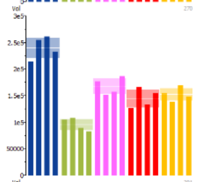	Seed maturation/ nutrient reservoir activity
291	Vicilin-like seed storage protein	AHYPO_006304-RA	VCL22_ARATH	22.8/7.6	61.9/5.9	391	6/14	0.42	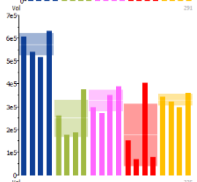	Seed maturation/ nutrient reservoir activity
335	Vicilin-like seed storage protein	AHYPO_010140-RA	VCL21_ARATH	26.7/5.9	67.2/5.4	171	4/5	0.25	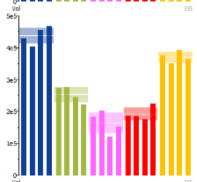	Seed maturation/ nutrient reservoir activity
345	Vicilin-like seed storage protein At2g18540	AHYPO_010140-RA	VCL21_ARATH	28.3/6.3	67.2/5.4	154	4/8	0.26	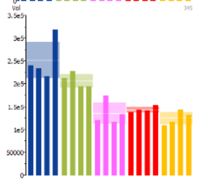	Seed maturation/ nutrient reservoir activity
348	Peroxygenase	AHYPO_016889-RA	PXG_SESIN	29.4/5.7	26.1/5.5	331	6/30	1.61		Lipid storage
	Granule-bound starch synthase I, chloroplastic/amyloplastic	AHYPO_011500-RA	SSG1_MANES		62.7/6.5	134	3/6	0.19		

	Triosephosphate isomerase, cytosolic	AHYPO_017821-RA	TPIS_COPJA		18.7/5.2	120	3/20	0.76	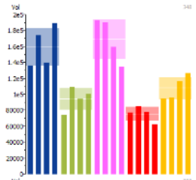	
353	Oil body-associated protein 1A	AHYPO_009953-RA	OBP1A_ARATH	31.1/7.0	26.6/6.2	130	3/16	0.66	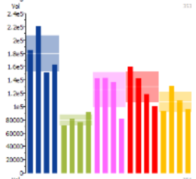	Lipid storage
354	Oil body-associated protein 1A	AHYPO_009953-RA	OBP1A_ARATH	30.6/6.8	26.6/6.2	387	10/52	3.92	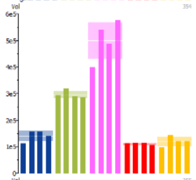	Lipid storage
355	Oil body-associated protein 1A	AHYPO_009953-RA	OBP1A_ARATH	29.7/6.7	26.6/6.2	159	2/11	0.51	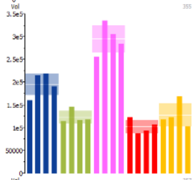	Lipid storage
	Proteasome subunit alpha type-6	AHYPO_008388-RA	PSA6_TOBAC		27.3/6.1	99	3/14	0.49		
357	Oil body-associated protein 1A	AHYPO_009953-RA	OBP1A_ARATH		26.6/6.2	379	6/33	1.65	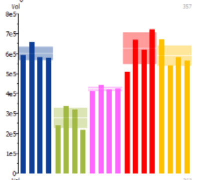	Lipid storage
	11S globulin	AHYPO_021282-RA	13SB_FAGES		77.6/7.0	149	2/3	0.10		
362	Vicilin-like seed storage protein	AHYPO_006304-RA	VCL22_ARATH	30.5/7.3	61.9/5.9	112	3/8	0.20	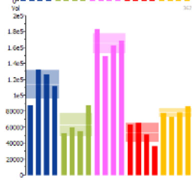	Seed maturation/ nutrient reservoir activity

365	Peroxygenase	AHYPO_016889-RA	PXG_SESIN	31.0/6.0	26.1/5.5	184	4/21	1.01	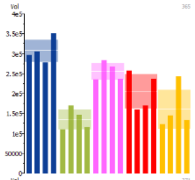	Lipid storage
371	Vicilin-like seed storage protein	AHYPO_006304-RA	VCL22_ARATH	31.5/5.3	61.9/5.9	184	4/9	0.28	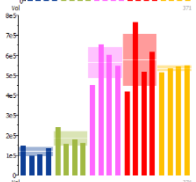	Seed maturation/ nutrient reservoir activity
374	Vicilin-like seed storage protein	AHYPO_006304-RA	VCL22_ARATH	31.7/5.3	61.9/5.9	633	5/11	0.44	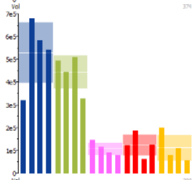	Seed maturation/ nutrient reservoir activity
390	11S globulin	AHYPO_001411-RA	CRU1_RAPSA	35.8/6.0	55.4/6.3	205	3/5	0.24	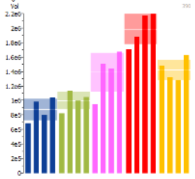	Seed maturation/ nutrient reservoir activity
402	Agglutinin	AHYPO_007409-RA	Q38719_AMAHP	35.8/7.0	30.1/6.5	641	5/27	1.00	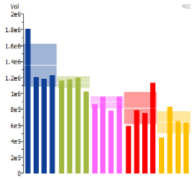	Carbohydrate binding
	11S globulin	AHYPO_021282-RA	13SB_FAGES		77.6/7.0	232	4/8	0.20		
	Glucose and ribitol dehydrogenase	AHYPO_010964-RA	GRDH_DAUCA		31.5/6.5	86	2/8	0.25		
404	Annexin-like protein RJ4	AHYPO_021089-RA	ANX4_FRAAN	36.6/7.3	24.3/7.8	219	7/30	2.16	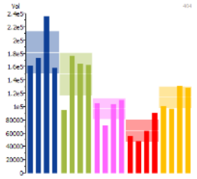	Stress response
	Agglutinin	AHYPO_007409-RA	Q38719_AMAHP		30.1/6.5	149	3/14	0.42		

405	11S globulin	AHYPO_021282-RA	13SB_FAGES	36.7/5.8	77.6/7.0	575	9/17	0.61	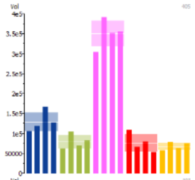	Seed maturation/ nutrient reservoir activity
408	Agglutinin	AHYPO_007409-RA	Q38719_AMAHP	38.9/6.7	30.1/6.5	131	3/14	0.42	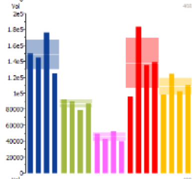	Carbohydrate binding
	Vicilin-like seed storage protein	AHYPO_018839-RA	AMP22_MACIN		60.9/6.6	71	3/6	0.19		
	Malate dehydrogenase, mitochondrial	AHYPO_004479-RA	MDHM_CITLA		36.2/8.4	57	2/7	0.21		
409	Agglutinin	AHYPO_007409-RA	Q38719_AMAHP	37.8/6.6	30.1/6.5	127	3/14	0.44	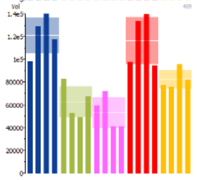	Carbohydrate binding
421	Vicilin-like seed storage protein	AHYPO_018839-RA	AMP22_MACIN	38.3/7.4	60.9/6.6	381	8/15	0.63	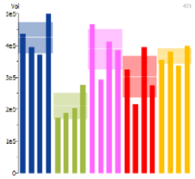	Seed maturation/ nutrient reservoir activity
	11S globulin	AHYPO_021282-RA	13SB_FAGES		77.6/7.0	132	2/4	0.10		
	Bifunctional UDP-glucose 4-epimerase and UDP-xylose 4-epimerase 1	AHYPO_012350-RA	UGE1_PEA		37.6/6.5	77	2/6	0.22		
422	60S acidic ribosomal protein P0-1	AHYPO_013703-RA	RLA01_ARATH	38.5/5.6	34.3/5.2	142	4/15	0.56	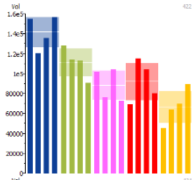	Translation
	11-beta-hydroxysteroid dehydrogenase 1B	AHYPO_004692-RA	HSD1B_ARATH		73.7/5.7	90	3/5	0.17		
424	Vicilin-like seed storage protein	AHYPO_018839-RA	AMP22_MACIN	38.7/7.3	60.9/6.6	602	9/18	0.79	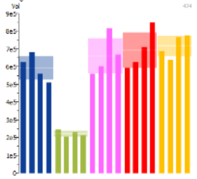	Seed maturation/ nutrient reservoir activity

425	Vicilin-like seed storage protein	AHYPO_018839-RA	AMP22_MACIN	38.9/7.3	60.9/6.6	720	8/15	0.69	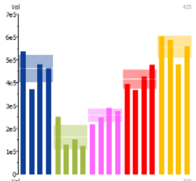	Seed maturation/ nutrient reservoir activity
429	Vicilin-like seed storage protein	AHYPO_018839-RA	AMP22_MACIN	39.2/6.6	60.9/6.6	246	5/10	0.42	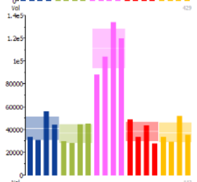	Seed maturation/ nutrient reservoir activity
	Agglutinin	AHYPO_007409-RA	Q38719_AMAHP		30.1/6.5	111	3/14	0.27		
	Annexin D2	AHYPO_020669-RA	ANXD2_ARATH		36.0/6.1	97	2/6	0.22		
442	Vicilin-like seed storage protein	AHYPO_018839-RA	AMP22_MACIN	40.5/7.0	60.9/6.6	252	6/13	0.47	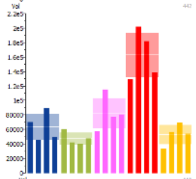	Seed maturation/ nutrient reservoir activity
	Agglutinin	AHYPO_007409-RA	Q38719_AMAHP		30.1/6.5	102	2/11	0.29		
443	Glyceraldehyde-3-phosphate dehydrogenase	AHYPO_011043-RA	G3P_ATRNU	39.3/7.6	31.6/6.7	395	10/36	2.58	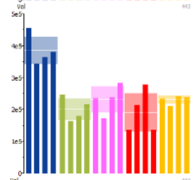	Glycolysis-TCA
444	Bifunctional UDP-glucose 4-epimerase and UDP-xylose 4-epimerase 1	AHYPO_012350-RA	UGE1_PEA	38.8/7.7	37.6/6.5	70	2/5	0.23	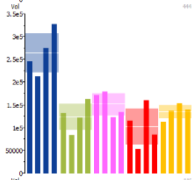	Cell wall-related
446	Glyceraldehyde-3-phosphate dehydrogenase	AHYPO_011043-RA	G3P_ATRNU	41.3/7.1	31.6/6.7	241	6/19	1.33	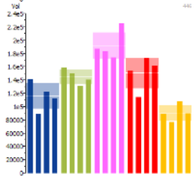	Glycolysis-TCA
451	Fructose-bisphosphate aldolase, cytoplasmic isozyme	AHYPO_000665-RA	ALF_SPIOL	41.2/6.8	38.3/6.2	203	4/18	0.51		Glycolysis-TCA

	Alcohol dehydrogenase 1	AHYPO_005892-RA	ADH1_PETHY		38.2/6.2	165	5/13	0.67	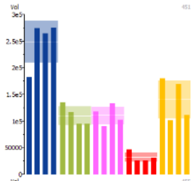	
455	11S globulin	AHYPO_021282-RA	13SB_FAGES	41.8/6.2	77.6/7.0	657	12/21	0.76	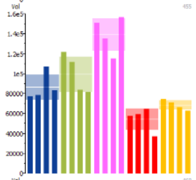	Seed maturation/ nutrient reservoir activity
460	11S globulin	AHYPO_021282-RA	13SB_FAGES	41.1/6.4	77.6/7.0	856	13/22	0.84	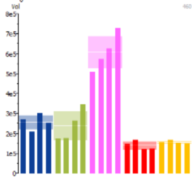	Seed maturation/ nutrient reservoir activity
472	11S globulin	AHYPO_021282-RA	13SB_FAGES	44.3/6.6	77.6/7.0	394	9/20	0.53	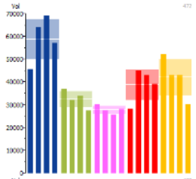	Seed maturation/ nutrient reservoir activity
	Alcohol dehydrogenase 1	AHYPO_005892-RA	ADH1_PETHY		38.2/6.2	149	2/5	0.21		
477	11S globulin	AHYPO_021282-RA	13SB_FAGES	47.3/6.7	77.6/7.0	571	13/22	1.00	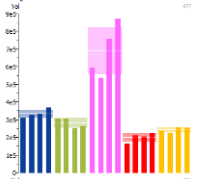	Seed maturation/ nutrient reservoir activity
481	11S globulin	AHYPO_021282-RA	13SB_FAGES	47.3/6.6	77.6/7.0	439	9/14	0.51	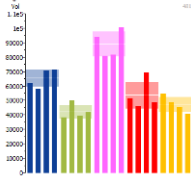	Seed maturation/ nutrient reservoir activity
491	S-adenosylmethionine synthase 4	AHYPO_018844-RA	METK4_ATRNU	49.6/5.9	43.3/5.5	448	8/26	1.06		Amino acids metabolism

	Granule-bound starch synthase I, chloroplastic/amyloplastic	AHYPO_011500-RA	SSG1_MANES		62.7/6.5	116	4/8	0.25	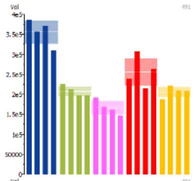	
	Caffeine synthase 1	AHYPO_013708-RA	TCS1_CAMSI		37.7/5.7	75	3/11	0.32		
494	11S globulin	AHYPO_021282-RA	13SB_FAGES	52.1/7.1	77.6/7.0	181	4/9	0.22	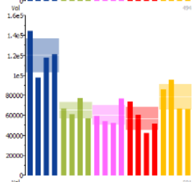	Seed maturation/ nutrient reservoir activity
501	11S globulin	AHYPO_021282-RA	13SB_FAGES	52.9/7.0	77.6/7.0	465	12/21	0.76	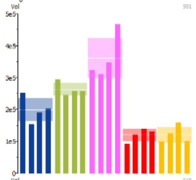	Seed maturation/ nutrient reservoir activity
518	11S globulin	AHYPO_021282-RA	13SB_FAGES	58.9/7.4	77.6/7.0	944	14/26	1.03	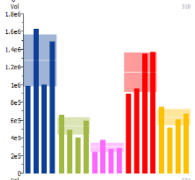	Seed maturation/ nutrient reservoir activity
	Serine hydroxymethyltransferase 4	AHYPO_009350-RA	GLYC4_ARATH		59.1/7.9	149	4/11	0.28		
524	11S globulin	AHYPO_021282-RA	13SB_FAGES	59.7/7.5	77.6/7.0	494	10/15	0.60	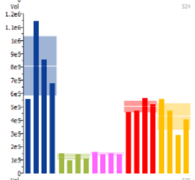	Seed maturation/ nutrient reservoir activity
	Serine hydroxymethyltransferase 4	AHYPO_009350-RA	GLYC4_ARATH		59.1/7.9	114	3/8	0.20		
525	Granule-bound starch synthase I, chloroplastic/amyloplastic	AHYPO_011500-RA	SSG1_MANES	60.2/6.5	62.7/6.5	101	2/3	0.12	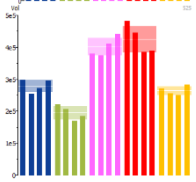	Starch biosynthetic process
	11S globulin	AHYPO_021282-RA	13SB_FAGES		77.6/7.0	77	2/4	0.10		

527	11S globulin	AHYPO_021282-RA	13SB_FAGES	60.0/7.3	77.6/7.0	672	13/21	0.84	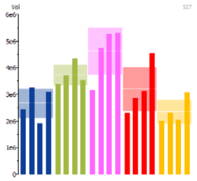	Seed maturation/ nutrient reservoir activity
530	11S globulin	AHYPO_021282-RA	13SB_FAGES	61.8/6.8	77.6/7.0	267	6/11	0.33	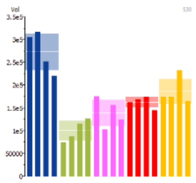	Seed maturation/ nutrient reservoir activity
	Vicilin-like seed storage protein	AHYPO_018839-RA	AMP22_MACIN		60.9/6.6	134	4/9	0.27		
	Granule-bound starch synthase I, chloroplastic/amyloplastic	AHYPO_011500-RA	SSG1_MANES		62.7/6.5	87	3/6	0.19		
533	11S globulin	AHYPO_021282-RA	13SB_FAGES	60.0/7.2	77.6/7.0	993	11/18	0.66	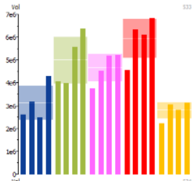	Seed maturation/ nutrient reservoir activity
534	11S globulin	AHYPO_021282-RA	13SB_FAGES	63.8/7.1	77.6/7.0	690	13/23	0.84	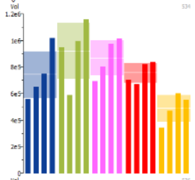	Seed maturation/ nutrient reservoir activity
536	11S globulin	AHYPO_021282-RA	13SB_FAGES	59.1/7.0	77.6/7.0	1184	12/22	0.88	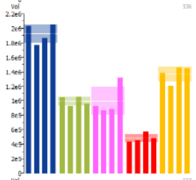	Seed maturation/ nutrient reservoir activity
537	11S globulin	AHYPO_021282-RA	13SB_FAGES	61.7/6.9	77.6/7.0	594	11/18	0.69	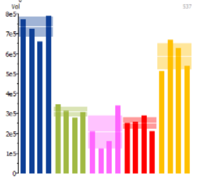	Seed maturation/ nutrient reservoir activity
	Granule-bound starch synthase I, chloroplastic/amyloplastic	AHYPO_011500-RA	SSG1_MANES		62.7/6.5	496	11/24	0.91		
	Vicilin-like seed storage protein	AHYPO_018839-RA	AMP22_MACIN		60.9/6.6	446	8/19	0.72		
541	11S globulin	AHYPO_021282-RA	13SB_FAGES	61.0/7.0	77.6/7.0	768	15/26	1.03		Seed maturation/ nutrient reservoir activity

	Granule-bound starch synthase I, chloroplastic/amyloplastic	AHYPO_011500-RA	SSG1_MANES		62.7/6.5	185	5/10	0.34	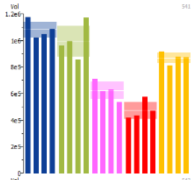	
	Vicilin-like seed storage protein	AHYPO_018839-RA	AMP22_MACIN		60.9/6.6	142	3/7	0.20		
543	11S globulin	AHYPO_021282-RA	13SB_FAGES		77.6/7.0	825	13/23	0.84	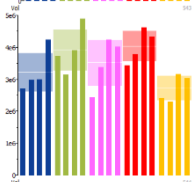	Seed maturation/ nutrient reservoir activity
544	11S globulin	AHYPO_021282-RA	13SB_FAGES	63.3/7.4	77.6/7.0	574	11/19	0.68	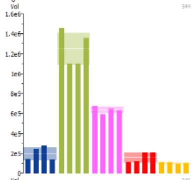	Seed maturation/ nutrient reservoir activity
546	11S globulin	AHYPO_021282-RA	13SB_FAGES	64.3/7.5	77.6/7.0	295	8/15	0.46	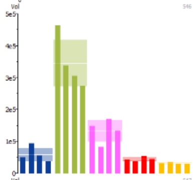	Seed maturation/ nutrient reservoir activity
	Granule-bound starch synthase I, chloroplastic/amyloplastic	AHYPO_011500-RA	SSG1_MANES		62.7/6.5	85	2/4	0.12		
547	Enolase	AHYPO_001182-RA	ENO_MESCR	63.4/5.9	48.2/5.5	159	4/13	0.36	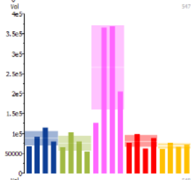	Glycolysis-TCA
548	11S globulin	AHYPO_021282-RA	13SB_FAGES	64.0/7.5	77.6/7.0	565	11/21	0.68	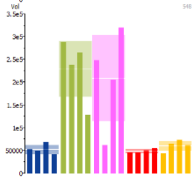	Seed maturation/ nutrient reservoir activity

549	Granule-bound starch synthase I, chloroplastic/amyloplastic	AHYPO_011500-RA	SSG1_MANES	62.3/6.4	62.7/6.5	1279	16/38	2.23	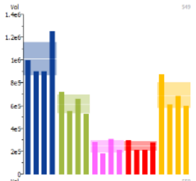	Starch biosynthetic process
550	Granule-bound starch synthase I, chloroplastic/amyloplastic	AHYPO_011500-RA	SSG1_MANES	64.3/6.5	62.7/6.5	928	14/30	1.49	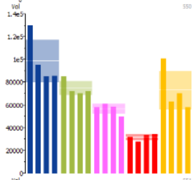	Starch biosynthetic process
551	Granule-bound starch synthase I, chloroplastic/amyloplastic	AHYPO_011500-RA	SSG1_MANES	63.5/6.3	62.7/6.5	1063	19/46	2.91	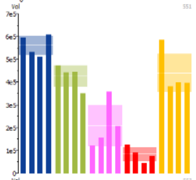	Starch biosynthetic process
553	Granule-bound starch synthase I, chloroplastic/amyloplastic	AHYPO_011500-RA	SSG1_MANES	64.4/6.5	62.7/6.5	986	17/36	1.82	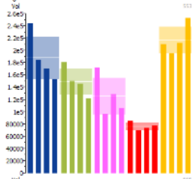	Starch biosynthetic process
565	Granule-bound starch synthase I, chloroplastic/amyloplastic	AHYPO_011500-RA	SSG1_MANES	71.4/7.6	62.7/6.5	992	19/40	2.28	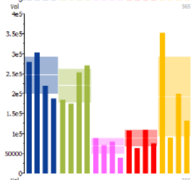	Starch biosynthetic process
566	Granule-bound starch synthase I, chloroplastic/amyloplastic	AHYPO_011500-RA	SSG1_MANES	71.4/7.5	62.7/6.5	846	16/35	1.56	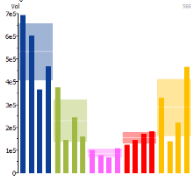	Starch biosynthetic process
	11S globulin	AHYPO_021282-RA	13SB_FAGES		77.6/7.0	353	8/12	0.46		
567	Granule-bound starch synthase I, chloroplastic/amyloplastic	AHYPO_011500-RA	SSG1_MANES	73.6/7.4	62.7/6.5	1106	17/36	1.88		Starch biosynthetic process

	11S globulin	AHYPO_021282-RA	13SB_FAGES		77.6/7.0	404	8/13	0.46	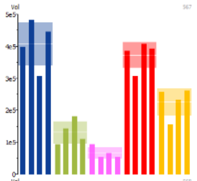	
568	Granule-bound starch synthase I, chloroplastic/amyloplastic	AHYPO_011500-RA	SSG1_MANES	74.7/7.1	62.7/6.5	1685	23/52	4.09	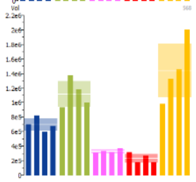	Starch biosynthetic process

[a]Spot number assigned by Melanie software. [b]Accession number according to the database reported by Clouse et al. 2016. [c]UniProtKB/Swiss-Prot ortholog identifier assigned by Trinotate. [d]Experimental mass and isoelectric point. [e]Theoretical mass and isoelectric point. [f]MASCOT Score, individual ion scores statistically significant at $p<0.001$, only identifications with peptide matches above the identity threshold when FDR≤1% were considered true. [g]Peptides Matched/Sequence Coverage. [h]Exponentially Modified Protein Abundance Index. [i]Protein spot accumulation change histograms ($p \leq 0.001$ and fold change≥2.0): A, *A. hybridus*; B, *A. powellii*; C, *A. cruentus* cv Amaranteca; D, *A. hypochondriacus* cv Opaca; E, *A. hypochondriacus* cv Cristalina. [j]The metabolic process of the main identified protein based on emPAI is shown.
